# Adherence to reporting guidelines increases the number of citations: the argument for including a methodologist in the editorial process and peer-review

**DOI:** 10.1186/s12874-019-0746-4

**Published:** 2019-05-31

**Authors:** Marta Vilaró, Jordi Cortés, Albert Selva-O’Callaghan, Agustín Urrutia, Josep-Maria Ribera, Francesc Cardellach, Xavier Basagaña, Matthew Elmore, Miquel Vilardell, Douglas Altman, José-Antonio González, Erik Cobo

**Affiliations:** 1 Universitat Politècnica Catalunya, Barcelona, Spain; 2 Medicina Clínica, Elsevier-Barcelona, Barcelona, Spain; 3 Universitat Autònoma de Barcelona, Barcelona, Spain; 4 Vall D’Hebron Hospital, Barcelona, Spain; 5 Hospital Germans Trias I Pujol, Badalona, Spain; 6 José Carreras Leukaemia Research Institute, Catalan Institute of Oncology, Badalona, Spain; 7 Universitat de Barcelona and Hospital Clínic, Barcelona, Spain; 8 ISGlobal, Centre for Research in Environmental Epidemiology (CREAL), Barcelona, Spain; 9 Universitat Pompeu Fabra, Barcelona, Spain; 10 Ciber Epidemiología y Salud Pública (CIBERESP), Barcelona, Spain; 11 Centre for Statistics in Medicine, University of Oxford, Botnar Research Centre, Oxford, UK; 12 Statistical Researcher, Statistics and Operational Research, Barcelona Tech, C/Jordi Girona, 1-3. Edifici C5, planta 2, Campus Nord, 08034 Barcelona, Spain

**Keywords:** Reporting guidelines, Peer-review, Reproducibility, Transparency, Number of citations

## Abstract

**Background:**

From 2005 to 2010, we conducted 2 randomized studies on a journal (Medicina Clínica), where we took manuscripts received for publication and randomly assigned them to either the standard editorial process or to additional processes. Both studies were based on the use of methodological reviewers and reporting guidelines (RG). Those interventions slightly improved the items reported on the Manuscript Quality Assessment Instrument (MQAI), which assesses the quality of the research report. However, masked evaluators were able to guess the allocated group in 62% (56/90) of the papers, thus presenting a risk of detection bias. In this post-hoc study, we analyse whether those interventions that were originally designed for improving the completeness of manuscript reporting may have had an effect on the number of citations, which is the measured outcome that we used.

**Methods:**

Masked to the intervention group, one of us used the Web of Science (WoS) to quantify the number of citations that the participating manuscripts received up December 2016. We calculated the mean citation ratio between intervention arms and then quantified the uncertainty of it by means of the Jackknife method, which avoids assumptions about the distribution shape.

**Results:**

Our study included 191 articles (99 and 92, respectively) from the two previous studies, which all together received 1336 citations. In both studies, the groups subjected to additional processes showed higher averages, standard deviations and annual rates. The intervention effect was similar in both studies, with a combined estimate of a 43% (95% CI: 3 to 98%) increase in the number of citations.

**Conclusions:**

We interpret that those effects are driven mainly by introducing into the editorial process a senior methodologist to find missing RG items. Those results are promising, but not definitive due to the exploratory nature of the study and some important caveats such as: the limitations of using the number of citations as a measure of scientific impact; and the fact that our study is based on a single journal. We invite journals to perform their own studies to ascertain whether or not scientific repercussion is increased by adhering to reporting guidelines and further involving statisticians in the editorial process.

**Electronic supplementary material:**

The online version of this article (10.1186/s12874-019-0746-4) contains supplementary material, which is available to authorized users.

## Background

The full progress of science relies on peer review, yet many have called into question the benefits of peer review [1–7]. In essence, critics assert that “studies have shown that peer reviewers were not able to appropriately detect errors, improve the completeness of reporting, or decrease the distortion of the study results” [8]. Nevertheless, the purposes for which Reporting Guidelines (RG) have been developed over the past two decades are to help authors, editors and peer reviewers check and improve the transparency of research studies while ensuring that papers are both accurate and complete [9–14]. According to the systematic review published by Bruce et al. (2016) [8], which we expand on in Section 7 of the Additional file 1, at least 23 randomized trials have studied some aspects of the peer review process, with the majority of them focusing on the quality of peer review as a surrogate outcome while only 3 [15–17] analysed the completeness of reporting as an outcome. Of these 3 trials that we previously conducted, only 2 [15, 16] found positive results regarding completeness of reporting — although only one of these reached statistical significance. Those studies were based on a partly subjective outcome, the Manuscript Quality Assessment Instrument (MQAI) [18], and there is evidence that evaluators could have successfully guessed which were in the intervention group [8]. Consequently, raters could have favoured the group receiving an additional intervention, thus raising the risk of detection bias. Therefore, we follow up on those studies here by taking advantage of the Web of Science [19] (WoS) to reassess those 2 trials by using the number of citations later received by those papers. We consider such a measured outcome to be impartial and fair, as it is naturally free from the risk of evaluation bias. The relationship between the completeness of a report and the number of citations has been previously studied, with promising though not statistically significant results having been found [20, 21]. We also previously explored this relationship with a shorter follow-up (SM, Sections 5 and 6).

## Methods

**Fig. 1:**
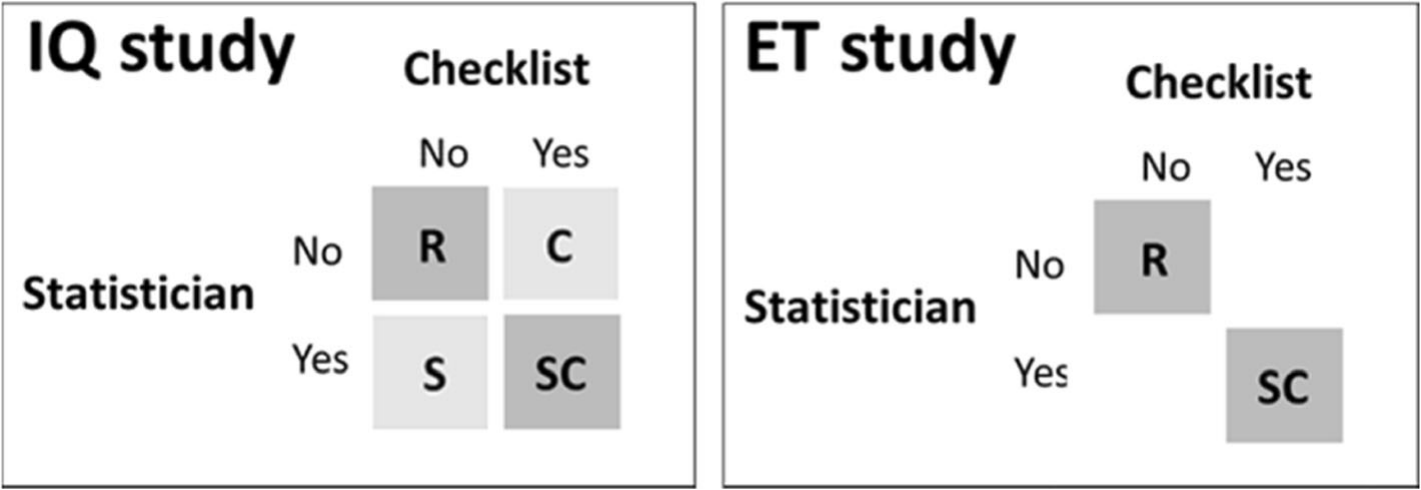
Scheme of the allocation of interventions of IQ and ET studies. Groups not included in the main analysis are in a shaded style. R = reference; C=Checklist; S=Statistician; SC = both Checklist and Statistician

We conducted two previous trials [15, 16], in which we found partially positive results from adding statistical reviewers and RGs to the peer review process. The first one was conducted in 2007 and called the “Improve Quality” (IQ) study [15], in which we randomly allocated 129 suitable manuscripts into 4 intervention groups (Fig. 1a). Unfortunately, after peer review, 16 manuscripts were rejected and 14 were lost to follow-up. Those losses introduced unpredictable (attrition) bias [22, 23] and may have affected the estimates.

The second trial was the 2011 “Enhance Transparency” (ET) study [16], in which we randomized 92 manuscripts either to both a statistical review and RGs or to neither (Fig. 1b). In both the IQ and ET studies, the main outcome was an assessed rather than measured endpoint. As masked evaluators were able to guess the intervention arm more often than could be ascribed to chance, partially unblinded evaluators could have introduced detection bias in both studies [8].

Due to these limitations, and in order to assess the long-term impact of those interventions, we adopted a new main outcome: the number of citations that each paper received on the WoS from publication up to December 312,016, with our hypothesis being that greater transparency and more comprehensible reporting may facilitate an increase in citations.

The IQ study divided the papers into 4 groups as a result of combining the two interventions into a 2 × 2 factorial design: a suggestion to the reviewers to employ an abridged checklist for the evaluation of basic biomedical research papers (C) [24]; and adding a statistician (S) from the reviewer panel list. Consequently, the 4 groups were defined as: papers which received a standard review process (reference), papers which received a review process using a local checklist (C), papers which received a standard review process and a revision from a statistician (S) and papers which received a standard review process and a revision from a statistician using a local checklist (SC). The reference intervention followed the usual journal process based on 1–3 reviewers. In order to combine those results with those of the ET study, only the 24 papers allocated to the group with both interventions (C and S) and the 27 allocated to the reference group (neither C nor S) were now included in the main analysis.

The ET study modified this design in 3 ways: first, by relying on just one senior methodological expert rather than choosing a statistical reviewer from an expert list; second, by combining both interventions, with the senior methodological reviewer proposing specific changes based on relevant international reporting guidelines; and, third, it avoided attrition by delaying the intervention until the decision had been made on whether or not to publish.

Masked to the intervention group, one of us (MV) collected from WoS the number of citations that the ET and IQ articles received. A search was made using the website’s search tab and including 3 references: (1) the publication name, “Medicina Clinica (Barcelona)”; (2) the publication year (either 2004 to 2005 or 2009 to 2010); and, (3) either the article’s title or by searching for the topic in order to consider posterior changes to the title (between the submitted and finally published version). Baseline MQAI and study group were obtained from the data of the ET and IQ studies.

We aim to estimate the ratio of the average citation-per-year between intervention arms (which we refer to in this paper as “mean citation ratio”). As the data did not fit to the distributional assumptions of the previously masked specified Poisson model, our main analysis relies on the more robust Jackknife method, which provides wider and more conservative intervals. As sensitivity analyses, we also report alternative analyses such as the previously mentioned Poisson model (Sections 2 to 4 of SM).

Additional collected variables are described in Section 1 of SM. Section 6 of SM and the master’s thesis of the first author [25] show the results of other exploratory data analyses that were previously performed with shorter follow-up.

Analyses were performed using R software version 3.2.1.

### Availability of data and materials

The dataset supporting the conclusions of this article is available at https://www-eio.upc.edu/redir/NumberCitations, where researchers can: (1) reproduce the results of our analysis; (2) check our data at the Web of Science [19] as of December 2016; and (3) update the number of citations in order to replicate our results with a longer follow-up. The critical scientist can try to reproduce both our outcome measurements and analyses.

## Results

**Fig. 2:**
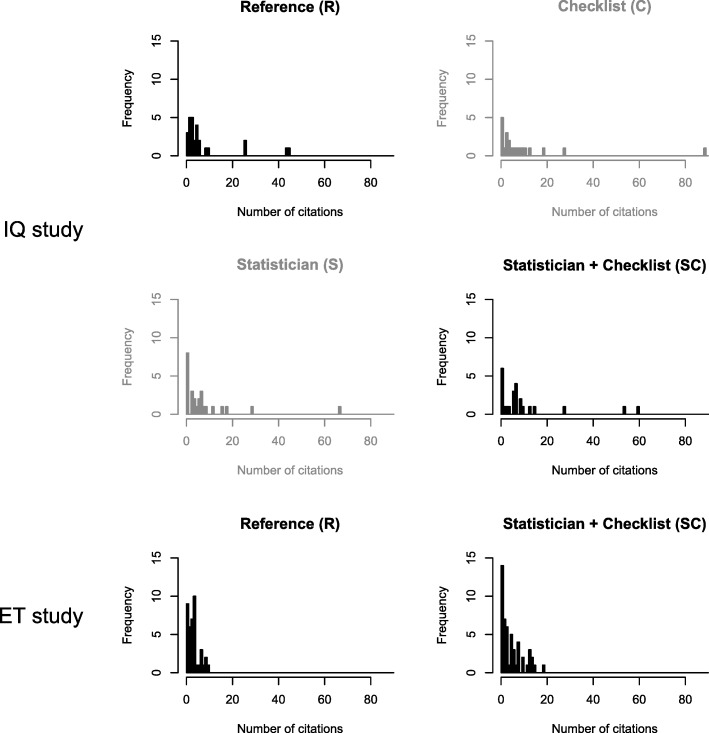
Number of citations by study and intervention group. Groups not included in the main analysis are in a shaded style

Of the 129 randomized papers, 99 IQ articles were published between 4 February 2005 and 12 May 2006, with a mean (standard deviation (SD)) follow-up period of 11.35 (0.31) years. Those publications received a total of 927 citations (mean 9.36, SD 14.87). ET included 92 randomized papers that were published between 24 June 2009 and 3 April 2010, with a mean (SD) follow-up period of 7.29 (0.31) years. They received a total of 409 citations (mean 4.44, SD 4.08). In both studies, the group with both interventions had larger means, standard deviations and annual rates. All intervention groups also had a slightly increased number of articles with 0 citations (Table 1 and Fig. 2).

**Table 1:** Number of citations by study and intervention group

				Number of citations		Annual rate	Articles with 0 citations
		N^1^	N^2^	Mean (SD)	Median (Max)	Mean (SD)	
IQ study	Standard review process (reference)	37	27	8.4 (12.2)	4 (45)	0.7 (1.1)	1 (3.7%)
	Statistician	31	26	8.4 (13.7)	4.5 (67)	0.7 (1.2)	4 (15.4%)
	Checklist	32	22	10.3 (18.8)	4.5 (89)	0.9 (1.6)	3 (13.6%)
	Statistician + Checklist	29	24	10.7 (15.5)	6.5 (60)	0.9 (1.3)	3 (12.5%)
ET study	Standard review process (reference)	41	41	3.6 (2.5)	3 (10)	0.5 (0.3)	2 (4.9%)
	Statistician + Checklist	51	51	5.1 (4.9)	3 (19)	0.7 (0.7)	7 (13.7%)

N^1^ = number of randomized manuscripts; N^2^ = number of analysed manuscripts

**Fig. 3:**
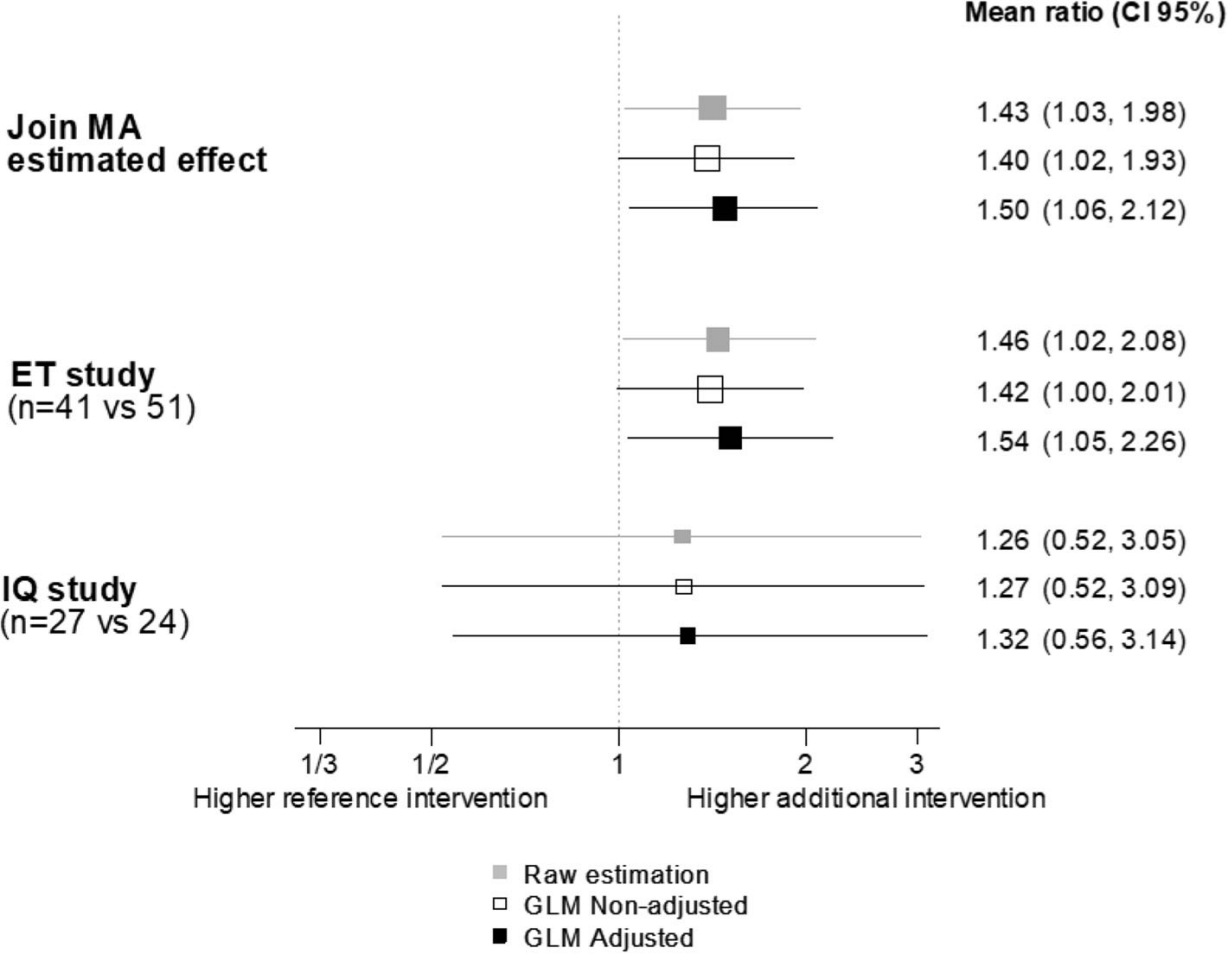
Citations-per-year mean ratio. Point effect estimates are obtained through (1) resampling methods with relaxed distribution assumptions; and generalized linear (GLM) Poisson Models using either (2) non-adjusted or (3) adjusted by follow-up methods. All _95%_CI estimates came from the Jackknife method

Figure 3 shows positive intervention effects that are similar in both studies. Combining both estimates, the intervention increased the citation rate by 43% (_95%_CI: 3 to 98%). This effect is due mainly to the ET study, which has higher weight (85.9) in the meta-analysis due to a more precise estimate. The weight of the studies within the meta-analysis has been calculated from the inverse of the variances of mean ratio estimates, thereby obtaining 31.58 and 5.17 for ET and IQ, respectively.

All analyses show some intervention effect (Fig. 3), which is slightly larger in the ET study while there is greater uncertainty from random error in the IQ study.

In order to check the robustness of the results, we ran sensitivity analyses: a pre-specified Poisson model (which provided shorter and consequently more-precise confidence intervals); and alternative statistical models that were suitable for count data (Sections 2 to 4 in SM). All together, these provided consistent results.

## Discussion

If we consider both studies together, we find that including a methodological reviewer (for example, a senior statistician) who is dedicated to looking for missing RG items increases the number of citations by 43% (_95%_CI: 3 to 98%), a result that — if this finding is sustained — might justify the cost and time expenditure by the journal [26, 27]. The number of papers with zero-citations was also higher in the intervention groups of both studies, which raises the possibility that greater transparency deters citations for some kinds of papers. This unexpected result warrants confirmation in future studies.

To the best of our knowledge, this is the first study showing that the completeness of reporting is potentially associated with higher citation counts as a result of a specific intervention, namely: adding to the peer review process a methodological expert who ensures that the reporting guidelines are adhered to. Although the number of citations is considered by some authors to be an indicator of a paper’s influence [28–32], some have argued that “citation counts are an indicator more of impact than of quality or importance” [28]; thus, we should not conflate the number of citations with research quality [21, 33]. Due to the high uncertainty behind the IQ study (including the risk of bias due to attrition) and the weight of the ET study when estimating the combined effects, our interpretation mainly follows the ET results in that the formal use of RGs at the end of the editorial phase, after peer review, leads to an increase in the papers’ potential scientific value. This interpretation assumes that all added citations are “positive” in the sense that they contribute to the body of knowledge.

In interpreting this effect size, we should keep in mind the uncertainty reflected by the confidence intervals.

Our next important limitation pertains to the fact that our results rely on just one journal that is not top-quality and they therefore cannot be transported to top-tier journals where those interventions have probably already been implemented. According to the Scimago Journal Country Rank website, journals with Impact Factor ≥ 10 account for just 1% (15,259 out of 1,528,749 articles published in 2016) of biomedical scientific production; thus, our focus is not on the top-quality journals but on second-tier journals who could benefit from the intervention.

It is essential that our results be interpreted according to the exploratory nature of this extended follow-up study. First, we did not have enough advance information to know the fit between our data and the statistical models. Second, and more importantly, we had neither previous studies to sustain the hypothesis nor a sample size rationale to guarantee any desired power for testing this hypothesis. Therefore, in keeping with the American Statistical Association (ASA) statement on *p*-value [34], we should not interpret the results of any hypothesis test. Accordingly, we should also not be concerned about whether or not the 95% confidence intervals (CI) include the neutral value of 1, because there is no such previous hypothesis. However, as we stated prior to data collection that our objective is “to estimate the effects of those interventions on the number of citations”, selective outcome reporting is of no concern.

## Conclusions

Our findings indicate that the citation counts increased by 43% (95% CI from: 3 to 98%) after including in the editorial process a methodologist who ensures the proper reporting of checklist items. As our original studies were originally designed to test those hypotheses for a different outcome, this present study was not powered to test this post-hoc analysis; therefore, our results should not be interpreted as definitive and they need to be confirmed in properly powered designs. We invite journals to perform their own studies to ascertain whether or not scientific impact is increased, first, by adhering to reporting guidelines, and second, by further involving statisticians or methodological experts in the editorial process.

## Additional file

Additional file 1: Adherence to reporting guidelines increases the number of citations: the argument for including a methodologist in the editorial process and peer-review. Supplementary material. (DOCX 687 kb)

## Abbrevations

ASA: American Statistician Association
C: Intervention group with suggestion to the reviewers to employ an abridged checklist for the evaluation of basic biomedical research papers
CI: Confidence Interval
ET: “Enhance Transparency” study. Cobo E, Selva-O’Callaghan A, Ribera JM, Cardellach F, Dominguez R, Vilardell M. Statistical Reviewers Improve Reporting in Biomedical Articles: A Randomized Trial. Plos One. 2007; 2 (3): e332
IQ: “Improve Quality” study. Cobo E, Cortés J, Ribera JM, et al. Effect of using reporting guidelines during peer review on quality of final manuscripts submitted to a biomedical journal: masked randomized trial. BMJ. 2011; 343: d6783
MQAI: Manuscript Quality Assessment Instrument
RG: Reporting Guidelines
S: Intervention group adding a Statistician from the reviewer panel list statistician
SD: Standard Deviation
SM: Supplementary Material
WoS: Web of Science
