# Supplementary material for: Adherence to reporting guidelines increases the number of citations: the argument for including a methodologist in the editorial process and peer-review
Source: BMC Med Res Methodol. 2019 May 31;19:112. doi: 10.1186/s12874-019-0746-4 (PMC6544961; doi:10.1186/s12874-019-0746-4)
Supplement: Supplementary file 1 — Adherence to reporting guidelines increases the number of citations: the argument for including a methodologist in the editorial process and peer-review. Supplementary material. (DOCX 687 kb) [file 12874_2019_746_MOESM1_ESM.docx]

***Adherence to reporting guidelines increases the number of citations: the argument for including a methodologist in the editorial process and peer-review. Supplementary material***

Vilaró Marta, Cortés Jordi, Selva-O’Callaghan Albert, Urrutia Agustín, Ribera Josep María, Cardellach Francesc, Basagaña Xavier, Elmore Matthew, Vilardell Miquel, Altman Douglas G, González José Antonio, Cobo Erik

Contents

[SECTION 0	2](#_Toc10101299)

[SUPPLEMENTARY MATERIAL DESCRIPTION	2](#_Toc10101300)

[SECTION 1	3](#_Toc10101301)

[VARIABLES IN STUDY	3](#_Toc10101302)

[SECTION 2	4](#_Toc10101303)

[FIGURE OF MAIN ANALYSIS	4](#_Toc10101304)

[Statistician using Reporting Guidelines effect	4](#_Toc10101305)

[SECTION 3	5](#_Toc10101306)

[FIGURES OF SECONDARY ANALYSES:	5](#_Toc10101307)

[Statistician and Reporting Guidelines effects	5](#_Toc10101308)

[Statistician using Reporting Guidelines effect by Study type	7](#_Toc10101309)

[SECTION 4	8](#_Toc10101310)

[SENSITIVITY ANALYSES	8](#_Toc10101311)

[SECTION 5	11](#_Toc10101312)

[FIGURE OF ZERO-INFLATED POISSON MODEL	11](#_Toc10101313)

[SECTION 6	12](#_Toc10101314)

[EXPLORATORY ANALYSES OF ET STUDY	12](#_Toc10101315)

[1.1	Descriptive	12](#_Toc10101316)

[1.1.1	Univariate	12](#_Toc10101317)

[1.1.2	Bivariant	13](#_Toc10101318)

[1.1.3	By study Type	18](#_Toc10101319)

[1.2	Inference	19](#_Toc10101320)

[1.2.1	Comparison between groups (ITT)	19](#_Toc10101321)

[SECTION 7	21](#_Toc10101322)

[BIBLIOGRAPHIC SEARCH	21](#_Toc10101323)

[SECTION 8	21](#_Toc10101324)

[R Code	21](#_Toc10101325)

# SECTION 0

**SUPPLEMENTARY MATERIAL DESCRIPTION**

Supplementary material (SM) is divided into the 8 sections described below. Section 1 of this supplementary material describes the collected variables for the study. Section 2 shows previous specified (masked) parametric analyses that provided similar estimates of the effect but much smaller measures of uncertainty, resulting in overly narrow confidence intervals. Section 3 of SM presents the results of other possible comparisons in the IQ study, showing an intermediate numberof citations for the groups that had only one of the 2 interventions. The papers in the group with only RGs added have more citations than those with only the statistician reviewer added. The sensitivity analysis presented in Section 4 of SM shows that the effects are reasonably homogeneous over time, suggesting that our results also apply to the first two years. What is different here in comparison to the usual clinical studies, updating counts is relatively cheaper and we therefore did this three times previously: in 2014, to prepare a presentation for the CONSORT group (Section 6); in 2015 for the master’s dissertation of the first author (http://hdl.handle.net/2099.1/26123); and in April 2016 for a previous draft of the present results (Section 5). Our objective has always been to estimate the effects on citations; however, when updating the counts of 2015, a possible effect of increased variability was observed. Furthermore, when updating the counts in April 2016, we also updated the analysis to a zero-inflated Poisson model that could estimate 2 different effects: one on the percentage of papers with zero citations; and one on the change in the mean of citations. In the final look (updating counts since December 312,016), the number of zero-citations was smaller, thus making the zero-inflated Poisson model less necessary. In those models, to account for the different lengths of follow-up in the analyses, the time from publication was set as an offset variable. Section7 describes our bibliographic search to find all randomized controlled trials (RCTs) evaluating the impact of interventions to improve the quality of peer review in biomedical journals. This search was based on the systematic review of Bruce et al., which we expand on with a longer follow-up. We also searched for RCTs that had been experimentally assessed to improve the number of citations. Section 8 includes theR code used for the analyses.

# SECTION 1

**VARIABLES IN STUDY**

The next table describes the variables collected from the web of science for both studies.

| **Variable** | **Description** |
| --- | --- |
| **Title** | Article title |
| **Study** | Abbreviation name of the study where the article belongs (ET or IQ) |
| **Group** | Allocation treatment group |
| **Publication date** | Publication date of the article |
| **Follow-up** | Time from publication date to last update (26 April 2016) |
| **Goodman Scale** | Baseline Goodman Scale obtained in ET and IQ studies |
| **Number of citations** | Number of citations of the article |

# SECTION 2

**Counts updated since 31 December 2016.**

**FIGURE OF MAIN ANALYSIS**

**Statistician using Reporting Guidelines effect**

**Figure SM1**. Citations-per-year mean ratio obtained from the Poisson Model. Point effect estimates are obtained through (1) assumption-free resampling methods; and generalized linear (GLM) both (2) non-adjusted and (3) adjusted methods. GLM estimations with 95% CI coming from the Wald method.

# SECTION 3

**Counts updated since 31 December 2016.**

**FIGURES OF SECONDARY ANALYSES:**

**Statistician and Reporting Guidelines effects**

**Figure SM2**. Citations-per-year mean ratio for Statistician and Reporting Guidelines effects (IQ study only) obtained from raw estimation. Point effect estimates and 95%CI are obtained through assumption-free resampling methods.

**Figure SM3**. Citations-per-year mean ratio for Statistician and Reporting Guidelines effects (IQ study only) obtained from the Poisson Model. Point effect estimates are obtained through a generalized linear model (GLM) (1) non-adjusted and (2) adjusted methods. 95%CI coming from the more robust Jackknife method.

**Figure SM4**. Citations-per-year mean ratio for Statistician and Reporting Guidelines effects (IQ study only) obtained from the Poisson Model. Point effect estimates are obtained through a generalized linear model (GLM) (1) non-adjusted and (2) adjusted methods. 95% CI coming from the Wald method.

**Statistician using Reporting Guidelines effect by Study type**

**Figure SM5**. Citations-per-year mean ratio by Study type obtained from raw estimation. Point effect estimates and 95%CI are obtained through assumption-free resampling methods.

# SECTION 4

**Counts updated since 31 December 2016.**

**SENSITIVITY ANALYSES**

**Figure SM6.** Citations-per-year mean ratio obtained from other possible models (Quasi-Poisson model, Negative Binomial model and Zero-Inflated Negative Binomial model). Point effect estimates and 95%CI are obtained through assumption-free resampling methods.

**Figure SM7.** Cumulative Distribution Functions (CDFs) for Zero-Inflated Poisson, Poisson, Quasi-Poisson, Negative Binomial and Zero-Inflated Negative Binomial models to compare the adjustment of these different models. ET study.

**Figure SM8.** Cumulative Distribution Functions (CDFs) for Zero-Inflated Poisson, Poisson, Quasi-Poisson, Negative Binomial and Zero-Inflated Negative Binomial models to compare the adjustment of these different models. IQ study.

**Table SM1.** Crude comparison of the mean counts and the proportion of zeros P(0), reporting 95% CI of the mean difference and the difference of the proportion of zeros.

|  | | **Mean** | **95% CI mean difference** | **Proportion of zeros P(0)** | **95% CI difference of P(0)** |
| --- | --- | --- | --- | --- | --- |
| **ET study** | **R** | 3.6 | -3.1 to 0.1 | 4.9% | -97.3% to 79.6% |
|  | **SC** | 5.1 |  | 13.7% |  |
| **IQ study** | **R** | 8.4 | -10.2 to 5.7 | 3.7% | -92.2% to 74.6% |
|  | **SC** | 10.7 |  | 12.5% |  |
|  | **R+S** | 8.4 | -8.2 to 4.0 | 9.4% | -94.3% to 87.4% |
|  | **C+SC** | 10.5 |  | 13.0% |  |
|  | **R+C** | 9.2 | -7.4 to 5.2 | 8.2% | -98.3% to 86.6% |
|  | **S+SC** | 9.5 |  | 14.0% |  |
|  | **R** | 8.4 | -7.3 to 4.7 | 3.7% | -97.6% to 77.2%% |
|  | **S+C+SC** | 9.7 |  | 13.9% |  |
| R: Reference group (n=27); S: Statistician (n=26); C: Checklist (n=22); CS: Statistician+Checklist (n=24) | | | | | |

# SECTION 5

**Counts updated since 26 April 2014.**

**FIGURE OF ZERO-INFLATED POISSON MODEL**

**Figure SM9**. Mean ratio of citations (left) and odds ratio of extra-zeros (right). Empty and filled boxes represent direct and adjusted by baseline MQAI quality and length of follow up estimates. Uncertainty is estimated theoretically (Wald) and by resampling methodology (Jackknife).

# SECTION 6

**Counts updated until 31 April 2014.**

**EXPLORATORY ANALYSES OF ET STUDY**

## 1.1 Descriptive

### 1.1.1 Univariate

Sample of 92 papers published between January 24^th^, 2009 and April 3^rd^, 2010:

- 51 randomly assigned to statistical review based on RG
- 41 randomly assigned to conventional peer review (4 of them were finally treated as papers allocated in the other group)

Summary statistics:

|  | **n** | **Mean** | **SD** | **Min** | **Q1** | **Median** | **Q3** | **Max** |
| --- | --- | --- | --- | --- | --- | --- | --- | --- |
| Cites | 92 | 2.8 | 2.8 | 0.0 | 1.0 | 2.0 | 4.0 | 12.0 |
| References | 90 | 21.3 | 12.7 | 0.0 | 10.0 | 25.0 | 29.8 | 57.0 |
| Impact Factor Sum | 92 | 5.8 | 6.7 | 0.0 | 1.0 | 3.2 | 8.8 | 31.8 |
| Months published | 90 | 55.4 | 3.6 | 48.6 | 53.2 | 55.3 | 58.4 | 63.1 |

|  |  |
| --- | --- |

### 1.1.2 Bivariant

#### 1.1.2.1 Relationship among new collected variables

Next figure shows dispersion plots among all combinations of the previous four variables. Only a relevant relationship arises between the sum of the impact factors sum (of 2012) and the number of quotes.

#### 1.1.2.2 Relationship among new collected variables and previous quality variables

Next figure show the relationship between the previous quality variables collected for the BMJ paper and the new two impact variables (number of quotes and sum of impact factors).

In next figure, regression lines are plotted for all cases (first row), stratified by allocated group (2n row) and stratified by study type (third row). Only, statistically significant slope was found for intervention studys taking as response the number of quotes as well as the sum of the impact factors.

**By subgroups**

#### 1.1.2.3 Difference between groups. ITT - Number of quotes

**Frequencies**

|  | **Proportion of cites (row %)** | | | | | | | | | | | |
| --- | --- | --- | --- | --- | --- | --- | --- | --- | --- | --- | --- | --- |
|  | **0** | **1** | **2** | **3** | **4** | **5** | **6** | **7** | **8** | **9** | **10** | **12** |
| Cites - Intervention | 25.5 | 17.6 | 11.8 | 11.8 | 3.9 | 3.9 | 11.8 | 2 | 2 | 5.9 | 2 | 2 |
| Cites - Control | 14.6 | 24.4 | 22 | 7.3 | 17.1 | 4.9 | 2.4 | 2.4 | 4.9 | 0 | 0 | 0 |

**Numerical indicators**

|  | **n** | **Mean** | **SD** | **Min** | **Q1** | **Median** | **Q3** | **Max** |
| --- | --- | --- | --- | --- | --- | --- | --- | --- |
| Cites - Intervention | 51 | 3.1 | 3.2 | 0.0 | 0.5 | 2.0 | 5.5 | 12.0 |
| Cites - Control | 41 | 2.5 | 2.1 | 0.0 | 1.0 | 2.0 | 4.0 | 8.0 |

#### 1.1.2.4 Difference between groups. ITT – Sum of Impact Factors of 2012

|  | **n** | **Mean** | **SD** | **Min** | **Q1** | **Median** | **Q3** | **Max** |
| --- | --- | --- | --- | --- | --- | --- | --- | --- |
| IF sum - Intervention | 51 | 5.7 | 7.0 | 0.0 | 0.0 | 3.1 | 9.0 | 31.8 |
| IF sum - Control | 41 | 5.9 | 6.3 | 0.0 | 1.4 | 3.7 | 6.7 | 25.6 |

### 1.1.3 By study Type

#### 1.1.3.1 Difference between groups. ITT - Number of quotes

Numerical indicators

| **Intervention** | | | | | | | | |
| --- | --- | --- | --- | --- | --- | --- | --- | --- |
|  | **n** | **Mean** | **SD** | **Min** | **Q1** | **Median** | **Q3** | **Max** |
| Cites - Intervention | 8 | 2.6 | 2.3 | 0.0 | 1.0 | 2.0 | 3.8 | 6.0 |
| Cites - Control | 8 | 1.9 | 1.5 | 0.0 | 1.0 | 2.0 | 2.0 | 5.0 |
| **Longitudinal** | | | | | | | | |
|  | **n** | **Mean** | **SD** | **Min** | **Q1** | **Median** | **Q3** | **Max** |
| Cites - Intervention | 18 | 2.3 | 2.5 | 0.0 | 0.0 | 2.0 | 3.0 | 9.0 |
| Cites - Control | 20 | 2.7 | 2.6 | 0.0 | 0.75 | 1.5 | 4.0 | 8.0 |
| **Cross-sectional** | | | | | | | | |
|  | **n** | **Mean** | **SD** | **Min** | **Q1** | **Median** | **Q3** | **Max** |
| Cites - Intervention | 17 | 4.8 | 3.8 | 0.0 | 1.0 | 5.0 | 8.0 | 12.0 |
| Cites - Control | 9 | 3.1 | 1.8 | 1.0 | 2.0 | 3.0 | 4.0 | 7.0 |
| **Other** | | | | | | | | |
|  | **n** | **Mean** | **SD** | **Min** | **Q1** | **Median** | **Q3** | **Max** |
| Cites - Intervention | 8 | 1.6 | 2.3 | 0.0 | 0.0 | 1.0 | 2.0 | 7.0 |
| Cites - Control | 4 | 2.0 | 1.4 | 1.0 | 1.0 | 1.5 | 2.5 | 4.0 |

#### 1.1.3.2 Difference between groups. ITT – Impact factor sum (2012)

Numerical indicators

| **Intervention** | | | | | | | | |
| --- | --- | --- | --- | --- | --- | --- | --- | --- |
|  | **n** | **Mean** | **SD** | **Min** | **Q1** | **Median** | **Q3** | **Max** |
| IF sum - Intervention | 8 | 4.8 | 4.9 | 0.0 | 1.5 | 3.3 | 6.1 | 13.1 |
| IF sum - Control | 8 | 2.3 | 3.2 | 0.0 | 1.7 | 3.0 | 4.0 | 10.0 |
| **Longitudinal** | | | | | | | | |
|  | **n** | **Mean** | **SD** | **Min** | **Q1** | **Median** | **Q3** | **Max** |
| IF sum - Intervention | 18 | 4.7 | 6.0 | 0.0 | 0.0 | 2.6 | 8.1 | 20.4 |
| IF sum - Control | 20 | 6.4 | 7.1 | 0.0 | 1.0 | 4.8 | 7.8 | 25.6 |
| **Cross-sectional** | | | | | | | | |
|  | **n** | **Mean** | **SD** | **Min** | **Q1** | **Median** | **Q3** | **Max** |
| IF sum - Intervention | 17 | 8.8 | 9.1 | 0.0 | 1.4 | 7.8 | 12.6 | 31.8 |
| IF sum - Control | 9 | 8.5 | 6.9 | 1.4 | 2.2 | 5.6 | 13.5 | 19.0 |
| **Other** | | | | | | | | |
|  | **n** | **Mean** | **SD** | **Min** | **Q1** | **Median** | **Q3** | **Max** |
| IF sum - Intervention | 8 | 2.3 | 2.9 | 0.0 | 0.0 | 1.0 | 3.7 | 7.5 |
| IF sum - Control | 4 | 2.9 | 2.1 | 1.4 | 1.4 | 2.2 | 3.7 | 5.8 |

## 1.2 Inference

### 1.2.1 Comparison between groups (ITT)

#### 1.2.1.1 Response: Number of quotes

Variances are statically significant, but they aren’t if we do the transformation x 🡪 log(1+x)

**Without transformation**

```
	F test to compare two variances

data:  resp by gr.inv
F = 2.1789, num df = 50, denom df = 40, p-value = 0.01226
alternative hypothesis: true ratio of variances is not equal to 1
95 percent confidence interval:
 1.189126 3.913965
sample estimates:
ratio of variances
          2.178934
```

**With transformation**

```
	F test to compare two variances

data:  log.resp by gr.inv
F = 1.6906, num df = 50, denom df = 40, p-value = 0.08851
alternative hypothesis: true ratio of variances is not equal to 1
95 percent confidence interval:
 0.9226138 3.0367504
sample estimates:
ratio of variances
          1.690582
```

There is no difference in the average number of quotes between groups

```
	Welch Two Sample t-test

data:  resp by gr
t = -0.9747, df = 87.669, p-value = 0.3324
alternative hypothesis: true difference in means is not equal to 0
95 percent confidence interval:
 -1.6466905  0.5629985
sample estimates:
mean in group 0 mean in group 1
       2.536585        3.078431
```

#### 1.2.1.2 Response: Impact factor sum (2012)

There is no difference in the average of sum of the impact factors between groups

```
	Two Sample t-test

data:  resp by gr
t = 0.1443, df = 90, p-value = 0.8856
alternative hypothesis: true difference in means is not equal to 0
95 percent confidence interval:
 -2.585515  2.990638
sample estimates:
mean in group 0 mean in group 1
       5.905463        5.702902
```

#### 1.2.1.3 Subgroups analysis

No differences were found in any subgroup type.

# SECTION 7

**BIBLIOGRAPHIC SEARCH**

Based on the systematic review published by Bruce et al^S7.1^ we search all randomized controlled trials (RCTs) evaluating the impact of interventions to improve the quality of peer review in biomedical journals.

They selected 22^S7.2-S7.23^ reports of randomized controlled trials, for 25 comparisons evaluating training interventions (n=5), the addition of a statistician peer reviewer (n=2), use of a checklist (n=2), open peer review (n=7), blinded peer review (n=6) and other interventions to increase the speed of the peer review process (n=3).

Following the same search methodology as in the review of Brue et al, an in order to expand it with longer follow-up, we searched for RCTs evaluating the impact of interventions aiming to improve the quality of peer review in biomedical journals published between 2015/06/15 and 2017/05/01 on the Cochrane Central Register of Controlled Trials (CENTRAL), MEDLINE (via PubMed), Embase, and the WHO International Clinical Trials Registry Platform for all reports of RCTs. We also searched the Cochrane Database of Systematic Reviews to identify systematic reviews on the peer review process. We had no limitation on language and our search strategy relied on the search term “peer review”. We hand-searched reference lists of reports and reviews dedicated to the peer review process identified during the screening process.

We only found 1^S7.24^ additional trial that studied changes in the peer review process:

References for section 7

[S7.1] Bruce R, Chauvin A, Trinquart L, Ravaud P, Boutron I. Impact of Interventions to Improve the Quality of Peer Review of Biomedical Journals: A Systematic Review and Meta-analysis. BMC Medicine (2016); 14:85.

[S7.2] Callaham ML, Knopp RK, Gallagher EJ. Effect of written feedback by editors on quality of reviews: two randomized trials. JAMA. 2002;287(21):2781–2783. doi: 10.1001/jama.287.21.2781.

[S7.3] Callaham ML, Schriger DL. Effect of structured workshop training on subsequent performance of journal peer reviewers. Ann Emerg Med. 2002;40(3):323–328. doi: 10.1067/mem.2002.127121.

[S7.4] Houry D, Green S, Callaham M. Does mentoring new peer reviewers improve review quality? A randomized trial. BMC Med Educ. 2012; 12:83. doi: 10.1186/1472-6920-12-83.

[S7.5] Schroter S, Black N, Evans S, et al. Effects of training on quality of peer review: randomised controlled trial. BMJ. 2004;328(7441):673. doi: 10.1136/bmj.38023.700775.AE.

[S7.6] Arnau C, Cobo E, Ribera JM, et al. [Effect of statistical review on manuscript quality in Medicina Clinica (Barcelona): a randomized study] Med Clin (Barc) 2003;121(18):690–694. doi: 10.1016/S0025-7753(03)74064-0.

[S7.7] Cobo E, Selva-O’Callagham A, Ribera JM, et al. Statistical reviewers improve reporting in biomedical articles: a randomized trial. PLoS One. 2007;2(3):e332. doi: 10.1371/journal.pone.0000332 Cobo E, Selva-O’Callagham A, Ribera JM, et al. Statistical reviewers improve reporting in biomedical articles: a randomized trial. PLoS One. 2007;2(3): e332. doi: 10.1371/journal.pone.0000332.

[S7.8] Cobo E, Cortes K, Ribera J, et al. Effect of using reporting guidelines during peer review on quality of final manuscripts submitted to a biomedical journal: masked randomised trial. BMJ. 2011; 343: d6783. doi: 10.1136/bmj. d6783.

[S7.9] Das Sinha S, Sahni P, Nundy S. Does exchanging comments of Indian and non-Indian reviewers improve the quality of manuscript reviews? Natl Med J India. 1999;12(5):210–213.

[S7.10] Godlee F, Gale CR, Martyn CN. Effect on the quality of peer review of blinding reviewers and asking them to sign their reports: a randomized controlled trial. JAMA. 1998;280(3):237–240. doi: 10.1001/jama.280.3.237.

[S7.11] Van Rooyen S, Godlee F, Evans S, et al. Effect of blinding and unmasking on the quality of peer review. JAMA. 1998;280(3):234–237. doi: 10.1001/jama.280.3.234.

[S7.12] Van Rooyen S, Godlee F, Evans S, et al. Effect of open peer review on quality of reviews and on reviewers’ recommendations: a randomised trial. BMJ. 1999; 318: 23–27. doi: 10.1136/bmj.318.7175.23.

[S7.13] Van Rooyen S, Delamothe T, Evans SJ. Effect on peer review of telling reviewers that their signed reviews might be posted on the web: randomised controlled trial. BMJ. 2010;341:c5729. doi: 10.1136/bmj.c5729.

[S7.14] Vinther S, Nielson OH, Rosenberg J, et al. Same review quality in open versus blinded peer review in “Ugeskrift for Laeger” Dan Med. 2012;59(8): A4479.

[S7.15] Walsh E, Rooney M, Appleby L, et al. Open peer review: a randomised trial. Br J Psychiatry. 2000; 176: 47–51. doi: 10.1192/bjp.176.1.47.

[S7.16] Alam M, Kim NA, Havey J, et al. Blinded vs. unblinded peer review of manuscripts submitted to a dermatology journal: a randomized multi-rater study. Br J Dermatol. 2011; 165: 563–567. doi: 10.1111/j.1365-2133.2011.10432.

[S7.17] Fisher M, Friedman SB, Strauss B. The effects of blinding on acceptance of research papers by peer review. JAMA. 1994;272(2):143–146. doi: 10.1001/jama.1994.03520020069019.

[S7.18] Godlee F, Gale CR, Martyn CN. Effect on the quality of peer review of blinding reviewers and asking them to sign their reports: a randomized controlled trial. JAMA. 1998;280(3):237–240. doi: 10.1001/jama.280.3.237.

[S7.19] Justice AC, Cho MK, Winker MA, et al. Does masking author identity improve peer review quality? PEER Investigators. JAMA. 1998;280(3):240–243. doi: 10.1001/jama.280.3.240.

[S7.20] McNutt RA, Evans AT, Fletcher RH, et al. The effects of blinding on the quality of peer review. A randomized trial. JAMA. 1990; 263(10): 1371–1376. doi: 10.1001/jama.1990.03440100079012.

[S7.21] Johnston SC, Lowenstein DH, Ferriero DM, et al. Early editorial manuscript screening versus obligate peer review: a randomized trial. Ann Neurol. 2007;61(4): A10–A12. doi: 10.1002/ana.21150.

[S7.22] Neuhauser D, Koran CJ. Calling medical care reviewers first: a randomized trial. Med Care. 1989;27(6):664–6.

[S7.23] Pitkin RM, Burmeister LF. Identifying manuscript reviewers: randomized comparison of asking first or just sending. JAMA. 2002;287(21):2795–2796. doi: 10.1001/jama.287.21.2795.

[S7.24] Okike K, Hug KT, Kocher MS, Leopold SS. Single-blind vs Double-blind Peer Review in the Setting of Author Prestige. *JAMA.* 2016;316(12):1315–1316. doi:10.1001/jama.2016.11014

# SECTION 8

**R Code**

```
########################################################################################
##
##-- ANALYSIS CODE
##-- Article: Adherence to Reporting Guidelines Increase Citations?
##
#######################################################################################

library(shiny)
library(rmeta)
library(pscl)

##-- Udated data since 31 December 2016
datos <- read.csv('data_actualitzat31122016.csv',header=TRUE,sep=";")


############----- FIGURES MAIN ARTICLE ----- ############

##############
##- Figure 2.
##############
##-- Histograms of the number of citations, by study group and by trial

windows()
par(mfrow=c(3,2))
hist(datos$NC[datos$Study=="ET" & datos$Group=="R"],ylim=c(0,15),breaks=c(0:90),xlab="Number of citations",main="Reference (R)",col="black")
hist(datos$NC[datos$Study=="ET" & datos$Group=="SC"],ylim=c(0,15),breaks=c(0:90),xlab="Number of citations",main="Statistician + Checklist (SC)",col="black")
hist(datos$NC[datos$Study=="IQ" & datos$Group=="R"],ylim=c(0,15),breaks=c(0:90),xlab="Number of citations",main="Reference (R)",col="black")
hist(datos$NC[datos$Study=="IQ" & datos$Group=="SC"],ylim=c(0,15),breaks=c(0:90),xlab="Number of citations",main="Statistician + Checklist (SC)",col="black")
hist(datos$NC[datos$Study=="IQ" & datos$Group=="S"],ylim=c(0,15),breaks=c(0:90),xlab="Number of citations",main="Statistician (S)",col="black")
hist(datos$NC[datos$Study=="IQ" & datos$Group=="C"],ylim=c(0,15),breaks=c(0:90),xlab="Number of citations",main="Checklist (C)",col="black")


##############
##- Figure 3.
##############
##-- Citations-per-year mean ratio obtained from the Poisson Model. Point effect estimates are obtained through (1) assumption-free resampling methods;
##-- and generalized linear (GLM) both (2) non-adjusted and (3) adjusted methods. All estimations with 95% CI coming from the more robust Jackknife methods

#- RAW ESTIMATION JACKKNIFE
log.mean.ratio <- function(dat) {d=dat; m0=mean(d$response[d$group=="R"],na.rm=T); m1=mean(d$response[d$group=="SC"],na.rm=T);log(m1/m0)} # Function to find log(mean ratio)
# ET study
response <- datos$NC[datos$Study=="ET"]/datos$Follow.up[datos$Study=="ET"];group <- datos$Group[datos$Study=="ET"]
dat <- data.frame(response,group=as.factor(group)); estim <- c(); estim[1] <- log.mean.ratio(dat)
for (i in 1:dim(dat)[1]){  d <- dat[-i,];  estim[i] <- log.mean.ratio(d)}
n <- dim(dat)[1]; desv <-sqrt(((n-1)/n)*sum((estim-mean(estim))^2)); estim.ET <- round(exp(mean(estim)),2); se.ET <- desv
LL.estim.ET <- round(exp(mean(estim)-qt(0.975,dim(datos[datos$Study=="ET",])[1]-1)*desv),2)
UL.estim.ET <- round(exp(mean(estim)+qt(0.975,dim(datos[datos$Study=="ET",])[1]-1)*desv),2)
# PLOS study: Both interventions effect (C vs CSK)
response <- datos$NC[datos$Study=="IQ" & (datos$Group=="R" | datos$Group=="SC")]/datos$Follow.up[datos$Study=="IQ" & (datos$Group=="R" | datos$Group=="SC")]
group <- datos$Group[datos$Study=="IQ" & (datos$Group=="R" | datos$Group=="SC")]; dat <- data.frame(response,group=as.factor(group))
estim <- c(); estim[1] <- log.mean.ratio(dat); for (i in 1:dim(dat)[1]){  d <- dat[-i,];  estim[i] <- log.mean.ratio(d)}
n <- dim(dat)[1]; desv <-sqrt(((n-1)/n)*sum((estim-mean(estim))^2)); estim.PLOS.1 <- round(exp(mean(estim)),2); se.PLOS.1 <- desv
LL.estim.PLOS.1 <- round(exp(mean(estim)-qt(0.975,dim(datos[datos$Study=="IQ" & (datos$Group=="R" | datos$Group=="SC"),])[1]-1)*desv),2)
UL.estim.PLOS.1 <- round(exp(mean(estim)+qt(0.975,dim(datos[datos$Study=="IQ" & (datos$Group=="R" | datos$Group=="SC"),])[1]-1)*desv),2)
# Meta-analysis
logs <- c(log(estim.ET),log(estim.PLOS.1)); selogs <- c(se.ET,se.PLOS.1); names <- c("ET study", "IQ study")
b <- data.frame(names,logs,selogs); d <- meta.summaries(b$logs, b$selogs, names=b$names, method="fix", logscale=TRUE)
estim.no.Adj.meta <- round(exp(d$summary),2); estim.no.Adj.meta.se <- round(d$se.summary,2)
LL.estim.no.Adj.meta <- round(summary(d)$summci[1],2); UL.estim.no.Adj.meta <- round(summary(d)$summci[3],2)
# Elements for the graphic
estim.ET.cru <- estim.ET; se.ET.cru <- se.ET; LL.estim.ET.cru <- LL.estim.ET; UL.estim.ET.cru <- UL.estim.ET
estim.PLOS.1.cru <- estim.PLOS.1; se.PLOS.1.cru <- se.PLOS.1; LL.estim.PLOS.1.cru <- LL.estim.PLOS.1; UL.estim.PLOS.1.cru <- UL.estim.PLOS.1
estim.no.Adj.meta.cru <- estim.no.Adj.meta; estim.no.Adj.meta.se.cru <- estim.no.Adj.meta.se; LL.estim.no.Adj.meta.cru <- LL.estim.no.Adj.meta
UL.estim.no.Adj.meta.cru <- UL.estim.no.Adj.meta
#- POISSON GLM JACKKNIFE
# ET: Adjusted Poisson (offset(FU) + Baseline Goodman scale)
S <- summary(m1 <- glm(NC ~ as.factor(Group)+offset(Follow.up)+Goodman.Scale, data = datos[datos$Study=="ET",],family="poisson")); estim <- c(); estim[1] <- S$coefficients[2,1]
for (i in 1:dim(datos[datos$Study=="ET",])[1]){d <- datos[datos$Study=="ET",][-i,]
S <- summary(m1 <- glm(NC ~ as.factor(Group)+offset(Follow.up)+Goodman.Scale, data = d,family="poisson")); estim[i] <- S$coefficients[2,1]}
n <- dim(datos[datos$Study=="ET",])[1]; desv <-sqrt(((n-1)/n)*sum((estim-mean(estim))^2)); ET_OR1 <- exp(mean(estim)); ET_OR1.pois_o <- ET_OR1; se.Adj.ET.pois <- desv
ET_OR1_inf <- exp(mean(estim)-qt(0.975,dim(datos[datos$Study=="ET",])[1]-1)*desv); ET_OR1_sup <- exp(mean(estim)+qt(0.975,dim(datos[datos$Study=="ET",])[1]-1)*desv)
d1_o <- data.frame(x=c("C vs CSK"),y=round(c(ET_OR1),2),ylo=round(c(ET_OR1_inf),2),yhi=round(c(ET_OR1_sup),2))
# ET: Non-Adjusted Poisson
S <- summary(m1 <- glm(NC ~ as.factor(Group), data = datos[datos$Study=="ET",],family="poisson")); estim <- c(); estim[1] <- S$coefficients[2,1]
for (i in 1:dim(datos[datos$Study=="ET",])[1]){d <- datos[datos$Study=="ET",][-i,]; S <- summary(m1 <- glm(NC ~ as.factor(Group), data = d,family="poisson"))
estim[i] <- S$coefficients[2,1]}
n <- dim(datos[datos$Study=="ET",])[1]; desv <-sqrt(((n-1)/n)*sum((estim-mean(estim))^2)); ET_OR1 <- exp(mean(estim)); ET_OR1.pois <- ET_OR1; se.no.Adj.ET <- desv
ET_OR1_inf <- exp(mean(estim)-qt(0.975,dim(datos[datos$Study=="ET",])[1]-1)*desv); ET_OR1_sup <- exp(mean(estim)+qt(0.975,dim(datos[datos$Study=="ET",])[1]-1)*desv)
d1 <- data.frame(x=c("C vs CSK"),y=round(c(ET_OR1),2),ylo=round(c(ET_OR1_inf),2),yhi=round(c(ET_OR1_sup),2))
# IQ: Adjusted Poisson (offset(FU) + Baseline Goodman scale)
articles1 <- subset(datos,Study=="IQ" & (Group=="R" | Group=="SC")); S <- summary(m1 <- glm(NC ~ as.factor(Group)+offset(Follow.up)+Goodman.Scale, data = articles1,family="poisson"))
estim <- c(); estim[1] <- S$coefficients[2,1];
for (i in 1:dim(articles1)[1]){d <- articles1[-i,]; S <- summary(m1 <- glm(NC ~ as.factor(Group)+offset(Follow.up)+Goodman.Scale, data = d,family="poisson"))
estim[i] <- S$coefficients[2,1]}
n <- dim(articles1)[1]; desv <-sqrt(((n-1)/n)*sum((estim-mean(estim))^2)); PLOS_C_CSK_OR1 <- exp(mean(estim)); PLOS_C_CSK_OR1.pois_o <- PLOS_C_CSK_OR1; se.Adj.PLOS.pois <- desv
PLOS_C_CSK_OR1_inf <- exp(mean(estim)-qt(0.975,dim(articles1)[1]-1)*desv); PLOS_C_CSK_OR1_sup <- exp(mean(estim)+qt(0.975,dim(articles1)[1]-1)*desv)
d4_o <- data.frame(x=c("C vs CSK"),y=round(c(PLOS_C_CSK_OR1),2),ylo=round(c(PLOS_C_CSK_OR1_inf),2),yhi=round(c(PLOS_C_CSK_OR1_sup),2))
# IQ: Non-Adjusted Poisson
S <- summary(m1 <- glm(NC ~ as.factor(Group), data = articles1,family="poisson")); estim <- c(); estim[1] <- S$coefficients[2,1]
for (i in 1:dim(articles1)[1]){d <- articles1[-i,]; S <- summary(m1 <- glm(NC ~ as.factor(Group), data = d,family="poisson")); estim[i] <- S$coefficients[2,1]}
n <- dim(articles1)[1]; desv <-sqrt(((n-1)/n)*sum((estim-mean(estim))^2)); PLOS_C_CSK_OR1 <- exp(mean(estim)); PLOS_C_CSK_OR1.pois <- PLOS_C_CSK_OR1; se.no.Adj.PLOS <- desv
PLOS_C_CSK_OR1_inf <- exp(mean(estim)-qt(0.975,dim(articles1)[1]-1)*desv); PLOS_C_CSK_OR1_sup <- exp(mean(estim)+qt(0.975,dim(articles1)[1]-1)*desv)
d4 <- data.frame(x=c("C vs CSK"),y=round(c(PLOS_C_CSK_OR1),2),ylo=round(c(PLOS_C_CSK_OR1_inf),2),yhi=round(c(PLOS_C_CSK_OR1_sup),2))
# Meta-analysis
# Unadjusted
ET <- rbind(d1,d1_o); PLOS_CvsCSK <- rbind(d4,d4_o); logs <- c(log(ET$y[1]),log(PLOS_CvsCSK$y[1])); selogs <- c(se.no.Adj.ET,se.no.Adj.PLOS)
names <- c("ET study", "IQ study"); b <- data.frame(names,logs,selogs); d <- meta.summaries(b$logs, b$selogs, names=b$names,method="fix", logscale=TRUE)
estim.no.Adj.meta <- round(exp(d$summary),2); LL.estim.no.Adj.meta <- round(summary(d)$summci[1],2); UL.estim.no.Adj.meta <- round(summary(d)$summci[3],2)
se.estim.meta.no.Adj <- d$se.summary
# Adjusted
logs <- c(log(ET$y[2]),log(PLOS_CvsCSK$y[2])); selogs <- c(se.Adj.ET.pois,se.Adj.PLOS.pois); names <- c("ET study", "IQ study"); b <- data.frame(names,logs,selogs)
d <- meta.summaries(b$logs, b$selogs, names=b$names,method="fix", logscale=TRUE); estim.Adj.meta <- round(exp(d$summary),2)
LL.estim.Adj.meta <- round(summary(d)$summci[1],2); UL.estim.Adj.meta <- round(summary(d)$summci[3],2); se.estim.meta.Adj <- d$se.summary
# Elements for the graphic
ET.jac <- ET; PLOS_CvsCSK.jac <- PLOS_CvsCSK; se.Adj.ET.jac <- se.Adj.ET.pois; se.no.Adj.ET.jac <- se.no.Adj.ET; se.Adj.PLOS.pois.jac <- se.Adj.PLOS.pois; se.no.Adj.PLOS.pois.jac <- se.no.Adj.PLOS;
estim.no.Adj.meta.jac <- estim.no.Adj.meta; LL.estim.no.Adj.meta.jac <- LL.estim.no.Adj.meta; UL.estim.no.Adj.meta.jac <- UL.estim.no.Adj.meta; se.estim.meta.no.Adj.jac <- se.estim.meta.no.Adj
estim.Adj.meta.jac <- estim.Adj.meta; LL.estim.Adj.meta.jac <- LL.estim.Adj.meta; UL.estim.Adj.meta.jac <- UL.estim.Adj.meta; se.estim.meta.Adj.jac <- se.estim.meta.Adj
# GRAHIC - Forest plot
win.graph(8,6); par(oma=c(0,6,0,9))
poin <- c(0,15); lin <- c(1,1); wid <- c(1,2); pos <- c(4:1); etiq <- c("(n=41 vs 51)","(n=27 vs 24)")
plot(NA, xlim = c(0.33,3), ylim=c(0,pos[1]),xaxt="n",yaxt="n",ylab="",xlab="",axes=F,log="x",main="",font.main=4)
abline(v=1,lty=3,col="darkgrey"); axis(1,c(1/3.5,0.33,0.5,1,2,3,3.5),labels=F); mtext(c("1/3","1/2","1","2","3"),las=1,side=1,at=c(0.33,0.5,1,2,3),line=0.5)
# MA
segments(LL.estim.Adj.meta.jac,pos[2],UL.estim.Adj.meta.jac,pos[2],lwd=wid[1],lty=1); points(estim.Adj.meta.jac,pos[2],pch=poin[2],cex=0.8/sqrt(se.estim.meta.Adj.jac))
segments(LL.estim.no.Adj.meta.jac,pos[2]+0.3,UL.estim.no.Adj.meta.jac,pos[2]+0.3,lwd=wid[1],lty=1); points(estim.no.Adj.meta.jac,pos[2]+0.3,pch=poin[1],cex=0.8/sqrt(se.estim.meta.no.Adj.jac))
segments(LL.estim.no.Adj.meta.cru,pos[2]+0.6,UL.estim.no.Adj.meta.cru,pos[2]+0.6,lwd=wid[1],col="darkgrey",lty=1); points(estim.no.Adj.meta.cru,pos[2]+0.6,pch=poin[2],cex=0.8/sqrt(estim.no.Adj.meta.se.cru),col="darkgrey")
# ET
segments(ET.jac$ylo[1],pos[3]-0.1,ET.jac$yhi[1],pos[3]-0.1,lwd=wid[1],lty=1); points(ET.jac$y[1],pos[3]-0.1,pch=poin[1],cex=0.8/sqrt(se.no.Adj.ET.jac))
segments(ET.jac$ylo[2],pos[3]-0.4,ET.jac$yhi[2],pos[3]-0.4,lwd=wid[1],lty=1); points(ET.jac$y[2],pos[3]-0.4,pch=poin[2],cex=0.8/sqrt(se.Adj.ET.jac))
segments(LL.estim.ET.cru,pos[3]+0.2,UL.estim.ET.cru ,pos[3]+0.2,lwd=wid[1],lty=1,col="darkgrey"); points(estim.ET.cru,pos[3]+0.2,pch=poin[2],cex=0.8/sqrt(se.ET.cru),col="darkgrey")
# IQ
segments(PLOS_CvsCSK.jac$ylo[1],pos[4]-0.3,PLOS_CvsCSK.jac$yhi[1],pos[4]-0.3,lwd=wid[1],lty=1); points(PLOS_CvsCSK.jac$y[1],pos[4]-0.3,pch=poin[1],cex=0.8/sqrt(se.no.Adj.PLOS.pois.jac))
segments(PLOS_CvsCSK.jac$ylo[2],pos[4]-0.6,PLOS_CvsCSK.jac$yhi[2],pos[4]-0.6,lwd=wid[1],lty=1); points(PLOS_CvsCSK.jac$y[2],pos[4]-0.6,pch=poin[2],cex=0.8/sqrt(se.Adj.PLOS.pois.jac))
segments(LL.estim.PLOS.1.cru ,pos[4],UL.estim.PLOS.1.cru ,pos[4],lwd=wid[1],lty=1,col="darkgrey"); points(estim.PLOS.1.cru,pos[4],pch=poin[2],cex=0.8/sqrt(se.PLOS.1.cru),col="darkgrey")
# Labels
mtext("Join MA",las=2,side=2,at=pos[2]+0.5,line=4.5,font=2,cex=1.2); mtext("estimated effect",las=2,side=2,at=pos[2]+0.3,line=0.8,font=2,cex=1.2)
mtext("ET study",las=2,side=2,at=pos[3],line=4,font=2,cex = 1.2); mtext(etiq[1],las=2,side=2,at=pos[3]-0.2,line=2.9,cex = 1.2)
mtext("IQ study",las=2,side=2,at=pos[4]-0.3,line=4.5,font=2,cex = 1.2); mtext(etiq[2],las=2,side=2,at=pos[4]-0.5,line=3.1,cex = 1.2)
mtext("Mean ratio (CI 95%)",las=2,side=4,at=pos[1]+0.2,line=0.7,font=2)
mtext(c("1.43","1.46","1.42","1.54","1.26","1.27","1.32"),side=4,las=2,at=c(3.6,2.2,1.9,1.6,1,0.7,0.4),line=1)
mtext(c("1.40","1.50"),side=4,las=2,at=c(3.3,3),line=1)
mtext(c("(1.03, 1.98)","(1.02, 2.08)","(0.52, 3.05)"),side=4,las=2,at=c(3.6,2.2,1),line=3,cex=1)
mtext(c("(1.02, 1.93)","(1.06, 2.12)","(1.00, 2.01)","(1.05, 2.26)","(0.52, 3.09)","(0.56, 3.14)"),side=4,las=2,at=c(3.3,3,1.9,1.6,0.7,0.4),line=3,cex=1)
mtext("Higher additional intervention",side=1,at=2.5,line=1.5); mtext("Higher reference intervention",side=1,at=1/2.5,line=1.5)


##########################----- FIGURES SUPPLEMENTARY MATERIAL ----- ######################

################
##- Figure SM1.
################
##-- Citations-per-year mean ratio obtained from the Poisson Model. Point effect estimates are obtained through (1) assumption-free resampling methods;
##-- and generalized linear (GLM) both (2) non-adjusted and (3) adjusted methods. All estimations with 95% CI coming from the Wald method.

#- RAW ESTIMATION JACKKNIFE
log.mean.ratio <- function(dat) {d=dat; m0=mean(d$response[d$group=="R"],na.rm=T); m1=mean(d$response[d$group=="SC"],na.rm=T);log(m1/m0)} # Function to find log(mean ratio)
# ET study
response <- datos$NC[datos$Study=="ET"]/datos$Follow.up[datos$Study=="ET"];group <- datos$Group[datos$Study=="ET"]
dat <- data.frame(response,group=as.factor(group)); estim <- c(); estim[1] <- log.mean.ratio(dat)
for (i in 1:dim(dat)[1]){  d <- dat[-i,];  estim[i] <- log.mean.ratio(d)}
n <- dim(dat)[1]; desv <-sqrt(((n-1)/n)*sum((estim-mean(estim))^2)); estim.ET <- round(exp(mean(estim)),2); se.ET <- desv
LL.estim.ET <- round(exp(mean(estim)-qt(0.975,dim(datos[datos$Study=="ET",])[1]-1)*desv),2)
UL.estim.ET <- round(exp(mean(estim)+qt(0.975,dim(datos[datos$Study=="ET",])[1]-1)*desv),2)
# PLOS study: Both interventions effect (C vs CSK)
response <- datos$NC[datos$Study=="IQ" & (datos$Group=="R" | datos$Group=="SC")]/datos$Follow.up[datos$Study=="IQ" & (datos$Group=="R" | datos$Group=="SC")]
group <- datos$Group[datos$Study=="IQ" & (datos$Group=="R" | datos$Group=="SC")]; dat <- data.frame(response,group=as.factor(group))
estim <- c(); estim[1] <- log.mean.ratio(dat); for (i in 1:dim(dat)[1]){  d <- dat[-i,];  estim[i] <- log.mean.ratio(d)}
n <- dim(dat)[1]; desv <-sqrt(((n-1)/n)*sum((estim-mean(estim))^2)); estim.PLOS.1 <- round(exp(mean(estim)),2); se.PLOS.1 <- desv
LL.estim.PLOS.1 <- round(exp(mean(estim)-qt(0.975,dim(datos[datos$Study=="IQ" & (datos$Group=="R" | datos$Group=="SC"),])[1]-1)*desv),2)
UL.estim.PLOS.1 <- round(exp(mean(estim)+qt(0.975,dim(datos[datos$Study=="IQ" & (datos$Group=="R" | datos$Group=="SC"),])[1]-1)*desv),2)
# Meta-analysis
logs <- c(log(estim.ET),log(estim.PLOS.1)); selogs <- c(se.ET,se.PLOS.1); names <- c("ET study", "IQ study")
b <- data.frame(names,logs,selogs); d <- meta.summaries(b$logs, b$selogs, names=b$names, method="fix", logscale=TRUE)
estim.no.Adj.meta <- round(exp(d$summary),2); estim.no.Adj.meta.se <- round(d$se.summary,2)
LL.estim.no.Adj.meta <- round(summary(d)$summci[1],2); UL.estim.no.Adj.meta <- round(summary(d)$summci[3],2)
# Elements for the graphic
estim.ET.cru <- estim.ET; se.ET.cru <- se.ET; LL.estim.ET.cru <- LL.estim.ET; UL.estim.ET.cru <- UL.estim.ET
estim.PLOS.1.cru <- estim.PLOS.1; se.PLOS.1.cru <- se.PLOS.1; LL.estim.PLOS.1.cru <- LL.estim.PLOS.1; UL.estim.PLOS.1.cru <- UL.estim.PLOS.1
estim.no.Adj.meta.cru <- estim.no.Adj.meta; estim.no.Adj.meta.se.cru <- estim.no.Adj.meta.se; LL.estim.no.Adj.meta.cru <- LL.estim.no.Adj.meta
UL.estim.no.Adj.meta.cru <- UL.estim.no.Adj.meta
# POISSON GLM WALD
# ET: Adjusted Poisson (offset(FU) + Baseline Goodman scale)
S <- summary(m1 <- glm(NC ~ as.factor(Group)+offset(Follow.up)+Goodman.Scale, data = datos[datos$Study=="ET",],family="poisson"))
estim <- S$coefficients[2,1]; desv <- S$coefficients[2,2]; ET_OR1 <- exp(estim); ET_OR1.pois_o <- ET_OR1; se.Adj.ET.pois <- desv
ET_OR1_inf <- exp(estim-qt(0.975,dim(datos)[1]-1)*desv); ET_OR1_sup <- exp(estim+qt(0.975,dim(datos)[1]-1)*desv)
d1_o <- data.frame(x=c("C vs CSK"),y=round(c(ET_OR1),2),ylo=round(c(ET_OR1_inf),2),yhi=round(c(ET_OR1_sup),2))
# ET: Non-Adjusted Poisson
S <- summary(m1 <- glm(NC ~ as.factor(Group), data = datos[datos$Study=="ET",],family="poisson"));
estim[1] <- S$coefficients[2,1]; desv <- S$coefficients[2,2]; ET_OR1 <- exp(estim); ET_OR1.pois <- ET_OR1; se.no.Adj.ET <- desv
ET_OR1_inf <- exp(estim-qt(0.975,dim(datos)[1]-1)*desv); ET_OR1_sup <- exp(estim+qt(0.975,dim(datos)[1]-1)*desv)
d1 <- data.frame(x=c("C vs CSK"),y=round(c(ET_OR1),2),ylo=round(c(ET_OR1_inf),2),yhi=round(c(ET_OR1_sup),2))
# IQ: Adjusted Poisson (offset(FU) + Baseline Goodman scale)
articles1 <- subset(datos,Study=="IQ" & (Group=="R" | Group=="SC"))
S <- summary(m1 <- glm(NC ~ as.factor(Group)+offset(Follow.up)+Goodman.Scale, data = articles1,family="poisson"))
estim[1] <- S$coefficients[2,1]; desv <- S$coefficients[2,2]; PLOS_C_CSK_OR1 <- exp(estim); PLOS_C_CSK_OR1.pois_o <- PLOS_C_CSK_OR1; se.Adj.PLOS.pois <- desv
PLOS_C_CSK_OR1_inf <- exp(estim-qt(0.975,dim(articles1)[1]-1)*desv); PLOS_C_CSK_OR1_sup <- exp(estim+qt(0.975,dim(articles1)[1]-1)*desv)
d4_o <- data.frame(x=c("C vs CSK"),y=round(c(PLOS_C_CSK_OR1),2),ylo=round(c(PLOS_C_CSK_OR1_inf),2),yhi=round(c(PLOS_C_CSK_OR1_sup),2))
# IQ: Non-Adjusted Poisson
S <- summary(m1 <- glm(NC ~ as.factor(Group), data = articles1,family="poisson")); estim[1] <- S$coefficients[2,1]; desv <- S$coefficients[2,2]
PLOS_C_CSK_OR1 <- exp(estim); PLOS_C_CSK_OR1.pois <- PLOS_C_CSK_OR1; se.no.Adj.PLOS <- desv
PLOS_C_CSK_OR1_inf <- exp(estim-qt(0.975,dim(articles1)[1]-1)*desv); PLOS_C_CSK_OR1_sup <- exp(estim+qt(0.975,dim(articles1)[1]-1)*desv)
d4 <- data.frame(x=c("C vs CSK"),y=round(c(PLOS_C_CSK_OR1),2),ylo=round(c(PLOS_C_CSK_OR1_inf),2),yhi=round(c(PLOS_C_CSK_OR1_sup),2))
# Meta-analysis
ET <- rbind(d1,d1_o); PLOS_CvsCSK <- rbind(d4,d4_o)
# Adjusted
logs <- c(log(ET$y[2]),log(PLOS_CvsCSK$y[2])); selogs <- c(se.Adj.ET.pois,se.Adj.PLOS.pois); names <- c("ET study", "IQ study")
b <- data.frame(names,logs,selogs); d <- meta.summaries(b$logs, b$selogs, names=b$names,method="fix", logscale=TRUE)
estim.Adj.meta <- round(exp(d$summary),2); LL.estim.Adj.meta <- round(summary(d)$summci[1],2); UL.estim.Adj.meta <- round(summary(d)$summci[3],2)
se.estim.meta.Adj <- d$se.summary
# Unadjusted
ET <- rbind(d1,d1_o); PLOS_CvsCSK <- rbind(d4,d4_o); logs <- c(log(ET$y[1]),log(PLOS_CvsCSK$y[1])); selogs <- c(se.no.Adj.ET,se.no.Adj.PLOS)
names <- c("ET study", "IQ study"); b <- data.frame(names,logs,selogs); d <- meta.summaries(b$logs, b$selogs, names=b$names,method="fix", logscale=TRUE)
estim.no.Adj.meta <- round(exp(d$summary),2); LL.estim.no.Adj.meta <- round(summary(d)$summci[1],2); UL.estim.no.Adj.meta <- round(summary(d)$summci[3],2)
se.estim.meta.no.Adj <- d$se.summary
# Elements for the graphic
ET.jac <- ET; PLOS_CvsCSK.jac <- PLOS_CvsCSK; se.Adj.ET.jac <- se.Adj.ET.pois; se.no.Adj.ET.jac <- se.no.Adj.ET; se.Adj.PLOS.pois.jac <- se.Adj.PLOS.pois; se.no.Adj.PLOS.pois.jac <- se.no.Adj.PLOS;
estim.no.Adj.meta.jac <- estim.no.Adj.meta; LL.estim.no.Adj.meta.jac <- LL.estim.no.Adj.meta; UL.estim.no.Adj.meta.jac <- UL.estim.no.Adj.meta; se.estim.meta.no.Adj.jac <- se.estim.meta.no.Adj
estim.Adj.meta.jac <- estim.Adj.meta; LL.estim.Adj.meta.jac <- LL.estim.Adj.meta; UL.estim.Adj.meta.jac <- UL.estim.Adj.meta; se.estim.meta.Adj.jac <- se.estim.meta.Adj
# GRAPHIc - Forest plot
win.graph(8,6); par(oma=c(0,6,0,9))
poin <- c(0,15); lin <- c(1,1); wid <- c(1,2); pos <- c(4:1); etiq <- c("(n=41 vs 51)","(n=27 vs 24)")
plot(NA, xlim = c(0.33,3), ylim=c(0,pos[1]),xaxt="n",yaxt="n",ylab="",xlab="",axes=F,log="x",main="",font.main=4)
abline(v=1,lty=3,col="darkgrey"); axis(1,c(1/3.5,0.33,0.5,1,2,3,3.5),labels=F); mtext(c("1/3","1/2","1","2","3"),las=1,side=1,at=c(0.33,0.5,1,2,3),line=0.5)
# MA
segments(LL.estim.Adj.meta.jac,pos[2],UL.estim.Adj.meta.jac,pos[2],lwd=wid[1],lty=1); points(estim.Adj.meta.jac,pos[2],pch=poin[2],cex=0.8/sqrt(se.estim.meta.Adj.jac))
segments(LL.estim.no.Adj.meta.jac,pos[2]+0.3,UL.estim.no.Adj.meta.jac,pos[2]+0.3,lwd=wid[1],lty=1); points(estim.no.Adj.meta.jac,pos[2]+0.3,pch=poin[1],cex=0.8/sqrt(se.estim.meta.no.Adj.jac))
segments(LL.estim.no.Adj.meta.cru,pos[2]+0.6,UL.estim.no.Adj.meta.cru,pos[2]+0.6,lwd=wid[1],col="darkgrey",lty=1); points(estim.no.Adj.meta.cru,pos[2]+0.6,pch=poin[2],cex=0.8/sqrt(estim.no.Adj.meta.se.cru),col="darkgrey")
# ET
segments(ET.jac$ylo[1],pos[3]-0.1,ET.jac$yhi[1],pos[3]-0.1,lwd=wid[1],lty=1); points(ET.jac$y[1],pos[3]-0.1,pch=poin[1],cex=0.8/sqrt(se.no.Adj.ET.jac))
segments(ET.jac$ylo[2],pos[3]-0.4,ET.jac$yhi[2],pos[3]-0.4,lwd=wid[1],lty=1); points(ET.jac$y[2],pos[3]-0.4,pch=poin[2],cex=0.8/sqrt(se.Adj.ET.jac))
segments(LL.estim.ET.cru,pos[3]+0.2,UL.estim.ET.cru ,pos[3]+0.2,lwd=wid[1],lty=1,col="darkgrey"); points(estim.ET.cru,pos[3]+0.2,pch=poin[2],cex=0.8/sqrt(se.ET.cru),col="darkgrey")
# IQ
segments(PLOS_CvsCSK.jac$ylo[1],pos[4]-0.3,PLOS_CvsCSK.jac$yhi[1],pos[4]-0.3,lwd=wid[1],lty=1); points(PLOS_CvsCSK.jac$y[1],pos[4]-0.3,pch=poin[1],cex=0.8/sqrt(se.no.Adj.PLOS.pois.jac))
segments(PLOS_CvsCSK.jac$ylo[2],pos[4]-0.6,PLOS_CvsCSK.jac$yhi[2],pos[4]-0.6,lwd=wid[1],lty=1); points(PLOS_CvsCSK.jac$y[2],pos[4]-0.6,pch=poin[2],cex=0.8/sqrt(se.Adj.PLOS.pois.jac))
segments(LL.estim.PLOS.1.cru ,pos[4],UL.estim.PLOS.1.cru ,pos[4],lwd=wid[1],lty=1,col="darkgrey"); points(estim.PLOS.1.cru,pos[4],pch=poin[2],cex=0.8/sqrt(se.PLOS.1.cru),col="darkgrey")
# Labels
mtext("Join MA",las=2,side=2,at=pos[2]+0.5,line=4.5,font=2,cex=1.2); mtext("estimated effect",las=2,side=2,at=pos[2]+0.3,line=0.8,font=2,cex=1.2)
mtext("ET study",las=2,side=2,at=pos[3],line=4,font=2,cex = 1.2); mtext(etiq[1],las=2,side=2,at=pos[3]-0.2,line=2.9,cex = 1.2)
mtext("IQ study",las=2,side=2,at=pos[4]-0.3,line=4.5,font=2,cex = 1.2); mtext(etiq[2],las=2,side=2,at=pos[4]-0.5,line=3.1,cex = 1.2)
mtext("Mean ratio (CI 95%)",las=2,side=4,at=pos[1]+0.2,line=0.7,font=2)
mtext(c("1.43","1.46","1.42","1.54","1.26","1.27","1.32"),side=4,las=2,at=c(3.6,2.2,1.9,1.6,1,0.7,0.4),line=1)
mtext(c("1.33","1.41"),side=4,las=2,at=c(3.3,3),line=1)
mtext(c("(1.03, 1.98)","(1.02, 2.08)","(0.52, 3.05)"),side=4,las=2,at=c(3.6,2.2,1),line=3,cex=1)
mtext(c("(1.17, 1.52)","(1.24, 1.62)","(1.16, 1.74)","(1.26, 1.89)","(1.06, 1.52)","(1.10, 1.59)"),side=4,las=2,at=c(3.3,3,1.9,1.6,0.7,0.4),line=3,cex=1)
mtext("Higher additional intervention",side=1,at=2.5,line=1.5); mtext("Higher reference intervention",side=1,at=1/2.5,line=1.5)


################
##- Figure SM2.
################
##-- Citations-per-year mean ratio for Statistician and Reporting Guidelines effects obtained from raw estimation.
##-- Point effect estimates and 95%CI are obtained through assumption-free resampling methods.

log.mean.ratio <- function(dat) {d=dat; m0=mean(d$response[d$group=="R"],na.rm=T); m1=mean(d$response[d$group=="T"],na.rm=T); log(m1/m0)} # Function to find log(mean ratio)
articles2 <- datos[datos$Study=="IQ",]
# STATISTICIAN
articles2$Group2[articles2$Group=="R" | articles2$Group=="C"] <- "R+C"
articles2$Group2[articles2$Group=="S" | articles2$Group=="SC"] <- "S+SC"
response <- articles2$NC/articles2$Follow.up
group <- articles2$Group2
group[group=="R+C"] <- "R"; group[group=="S+SC"] <- "T";
dat <- data.frame(response,group=as.factor(group))
estim <- c(); estim[1] <- log.mean.ratio(dat)
for (i in 1:dim(dat)[1]){  d <- dat[-i,];  estim[i] <- log.mean.ratio(d)}
n <- dim(dat)[1]; desv <-sqrt(((n-1)/n)*sum((estim-mean(estim))^2))
estim.ET <- round(exp(mean(estim)),2); se.ET <- desv
LL.estim.ET <- round(exp(mean(estim)-qt(0.975,dim(articles2)[1]-1)*desv),2)
UL.estim.ET <- round(exp(mean(estim)+qt(0.975,dim(articles2)[1]-1)*desv),2)
# REPORTING GUIDELINES
articles2$Group3[articles2$Group=="R" | articles2$Group=="S"] <- "R+S"
articles2$Group3[articles2$Group=="C" | articles2$Group=="SC"] <- "C+SC"
response <- articles2$NC/articles2$Follow.up
group <- articles2$Group3
group[group=="R+S"] <- "R"; group[group=="C+SC"] <- "T"
dat <- data.frame(response,group=as.factor(group))
estim <- c(); estim[1] <- log.mean.ratio(dat)
for (i in 1:dim(dat)[1]){  d <- dat[-i,];  estim[i] <- log.mean.ratio(d)}
n <- dim(dat)[1]; desv <-sqrt(((n-1)/n)*sum((estim-mean(estim))^2))
estim.PLOS.1 <- round(exp(mean(estim)),2); se.PLOS.1 <- desv
LL.estim.PLOS.1 <- round(exp(mean(estim)-qt(0.975,dim(articles2)[1]-1)*desv),2)
UL.estim.PLOS.1 <- round(exp(mean(estim)+qt(0.975,dim(articles2)[1]-1)*desv),2)
# GRAPHIC - Forest plot
poin <- c(0,15); lin <- c(1,1); wid <- c(1,2); pos <- c(2:1); etiq <- c("(n=49 vs 50)","(n=53 vs 46)")
win.graph(8,4); par(oma=c(0,8,0,7))
plot(NA, xlim = c(0.33,3), ylim=c(0,1.5),xaxt="n",yaxt="n", ylab="",xlab="",axes=F,log="x")
abline(v=1,lty=2); axis(1,c(1/3.5,0.33,0.5,1,2,3,3.5),labels=F)
mtext(c("1/3","1/2","1","2","3"),las=1,side=1,at=c(0.33,0.5,1,2,3),line=0.5)
# ET
segments(LL.estim.ET,1,UL.estim.ET,1,lwd=wid[1],col="darkgrey")
points(estim.ET,1,pch=poin[2],cex=1.34/sqrt(se.ET),col="darkgrey")
# IQ
segments(LL.estim.PLOS.1,0.3,UL.estim.PLOS.1,0.3,lwd=wid[1],length=0.1,col="darkgrey")
points(estim.PLOS.1,0.3,pch=poin[2],cex=1.34/sqrt(se.PLOS.1),col="darkgrey")
# Labels
mtext("Statistician",las=2,side=2,at=1,line=5.2,font=2,cex = 1.2)
mtext(etiq[1],las=2,side=2,at=0.8,line=5,cex = 1.2)
mtext("Reporting Guidelines",las=2,side=2,at=0.3,line=0.5,font=2,cex = 1.2)
mtext(etiq[2],las=2,side=2,at=0.1,line=5,cex = 1.2)
mtext("Mean ratio (CI 95%)",las=2,side=4,at=1.5,line=0.5,font=2)
mtext(paste("1.03","(","0.53",";","2.01",")"),side=4,las=2,at=1,line=1)
mtext(paste("1.24","(","0.64",";","2.40",")"),side=4,las=2,at=0.3,line=1)
mtext("Higher in intervention",las=2,side=2,at=-0.5,line=-21)
mtext("Higher in control",las=2,side=2,at=-0.5,line=-4)


################
##- Figure SM3.
################
##-- Citations-per-year mean ratio for Statistician and Reporting Guidelines effects (IQ study only) obtained from the Poisson Model.
##-- Point effect estimates are obtained through a generalized linear (GLM) (1) non-adjusted and (2) adjusted methods. All estimations
##-- with 95% CI coming from the more robust Jackknife method.

articles2 <- datos[datos$Study=="IQ",]
# STATISTICIAN
articles2$Group2[articles2$Group=="R" | articles2$Group=="C"] <- "R+C"
articles2$Group2[articles2$Group=="S" | articles2$Group=="SC"] <- "S+SC"
articles2$Group2 <- as.factor(articles2$Group2)
# Adjusted
S <- summary(m1 <- glm(NC ~ Group2+offset(Follow.up)+Goodman.Scale,data = articles2,family="poisson"));
estim <- c(); estim[1] <- S$coefficients[2,1]
for (i in 1:dim(articles2)[1]){d <- articles2[-i,]
S <- summary(m1 <- glm(NC ~ Group2+offset(Follow.up)+Goodman.Scale, data = d,family="poisson")); estim[i] <- S$coefficients[2,1]}
n <- dim(articles2)[1]; desv <-sqrt(((n-1)/n)*sum((estim-mean(estim))^2)); estim.ET <- exp(mean(estim)); se.ET <- desv
LL.estim.ET <- exp(mean(estim)-qt(0.975,dim(articles2)[1]-1)*desv); UL.estim.ET <- exp(mean(estim)+qt(0.975,dim(articles2)[1]-1)*desv)
# Unadjusted
S <- summary(m1 <- glm(NC ~ Group2,data = articles2,family="poisson"));
estim <- c(); estim[1] <- S$coefficients[2,1]
for (i in 1:dim(articles2)[1]){d <- articles2[-i,]
S <- summary(m1 <- glm(NC ~ Group2, data = d,family="poisson")); estim[i] <- S$coefficients[2,1]}
n <- dim(articles2)[1]; desv <-sqrt(((n-1)/n)*sum((estim-mean(estim))^2)); estim.ET.noadj <- exp(mean(estim)); se.ET.noadj <- desv
LL.estim.ET.noadj <- exp(mean(estim)-qt(0.975,dim(articles2)[1]-1)*desv); UL.estim.ET.noadj <- exp(mean(estim)+qt(0.975,dim(articles2)[1]-1)*desv)
# REPORTING GUIDELINES
articles2$Group3[articles2$Group=="R" | articles2$Group=="S"] <- "R+S"
articles2$Group3[articles2$Group=="C" | articles2$Group=="SC"] <- "C+SC"
articles2$Group3 <- as.factor(articles2$Group3)
articles2$Group3 <- relevel(articles2$Group3,ref="R+S")
# Adjusted
S <- summary(m1 <- glm(NC ~ as.factor(Group3)+offset(Follow.up)+Goodman.Scale,data = articles2,family="poisson"));
estim <- c(); estim[1] <- S$coefficients[2,1]
for (i in 1:dim(articles2)[1]){d <- articles2[-i,]
S <- summary(m1 <- glm(NC ~ as.factor(Group3)+offset(Follow.up)+Goodman.Scale, data = d,family="poisson")); estim[i] <- S$coefficients[2,1]}
n <- dim(articles2)[1]; desv <-sqrt(((n-1)/n)*sum((estim-mean(estim))^2)); estim.PLOS.1 <- exp(mean(estim)); se.PLOS.1 <- desv
LL.estim.PLOS.1 <- exp(mean(estim)-qt(0.975,dim(articles2)[1]-1)*desv); UL.estim.PLOS.1 <- exp(mean(estim)+qt(0.975,dim(articles2)[1]-1)*desv)
# Unadjusted
S <- summary(m1 <- glm(NC ~ as.factor(Group3),data = articles2,family="poisson"));
estim <- c(); estim[1] <- S$coefficients[2,1]
for (i in 1:dim(articles2)[1]){d <- articles2[-i,]
S <- summary(m1 <- glm(NC ~ as.factor(Group3), data = d,family="poisson")); estim[i] <- S$coefficients[2,1]}
n <- dim(articles2)[1]; desv <-sqrt(((n-1)/n)*sum((estim-mean(estim))^2)); estim.PLOS.1.noadj <- exp(mean(estim)); se.PLOS.1.noadj <- desv
LL.estim.PLOS.1.noadj <- exp(mean(estim)-qt(0.975,dim(articles2)[1]-1)*desv); UL.estim.PLOS.1.noadj <- exp(mean(estim)+qt(0.975,dim(articles2)[1]-1)*desv)
# GRAPHIC - Forest plot
poin <- c(0,15); lin <- c(1,1); wid <- c(1,2); pos <- c(2:1); etiq <- c("(n=49 vs 50)","(n=53 vs 46)")
win.graph(8,4); par(oma=c(0,8,0,7))
plot(NA, xlim = c(0.33,3), ylim=c(0,1.5),xaxt="n",yaxt="n", ylab="",xlab="",axes=F,log="x")
abline(v=1,lty=2); axis(1,c(1/3.5,0.33,0.5,1,2,3,3.5),labels=F)
mtext(c("1/3","1/2","1","2","3"),las=1,side=1,at=c(0.33,0.5,1,2,3),line=0.5)
# ET
segments(LL.estim.ET.noadj,1.2,UL.estim.ET.noadj,1.2,lwd=wid[1]); points(estim.ET.noadj,1.2,pch=poin[1],cex=1.34/sqrt(se.ET.noadj))
segments(LL.estim.ET,0.9,UL.estim.ET,0.9,lwd=wid[1]); points(estim.ET,0.9,pch=poin[2],cex=1.34/sqrt(se.ET))
# IQ
segments(LL.estim.PLOS.1.noadj,0.4,UL.estim.PLOS.1.noadj,0.4,lwd=wid[1]);points(estim.PLOS.1.noadj,0.4,pch=poin[1],cex=1.34/sqrt(se.PLOS.1.noadj))
segments(LL.estim.PLOS.1,0.1,UL.estim.PLOS.1,0.1,lwd=wid[1]);points(estim.PLOS.1,0.1,pch=poin[2],cex=1.34/sqrt(se.PLOS.1))
# Labels
mtext("Statistician",las=2,side=2,at=1,line=5.2,font=2,cex = 1.2)
mtext(etiq[1],las=2,side=2,at=0.8,line=5,cex = 1.2)
mtext("Reporting Guidelines",las=2,side=2,at=0.3,line=0.5,font=2,cex = 1.2)
mtext(etiq[2],las=2,side=2,at=0.1,line=5,cex = 1.2)
mtext("Mean ratio (CI 95%)",las=2,side=4,at=1.5,line=0.5,font=2)
mtext(paste("1.03","(","0.52",";","2.02",")"),side=4,las=2,at=1.2,line=1)
mtext(paste("1.00","(","0.49",";","2.05",")"),side=4,las=2,at=0.9,line=1)
mtext(paste("1.25","(","0.64",";","2.44",")"),side=4,las=2,at=0.4,line=1)
mtext(paste("1.28","(","0.65",";","2.52",")"),side=4,las=2,at=0.1,line=1)
mtext("Higher in intervention",las=2,side=2,at=-0.5,line=-21)
mtext("Higher in control",las=2,side=2,at=-0.5,line=-4)


################
##- Figure SM4.
################
##-- Citations-per-year mean ratio for Statistician and Reporting Guidelines effects (IQ study only) obtained from the Poisson Model.
##-- Point effect estimates are obtained through a generalized linear (GLM) (1) non-adjusted and (2) adjusted methods. All estimations
##-- with 95% CI coming from the Wald method.

articles2 <- datos[datos$Study=="IQ",]
# STATISTICIAN
articles2$Group2[articles2$Group=="R" | articles2$Group=="C"] <- "R+C"
articles2$Group2[articles2$Group=="S" | articles2$Group=="SC"] <- "S+SC"
articles2$Group2 <- as.factor(articles2$Group2)
# Adjusted
S <- summary(m1 <- glm(NC ~ Group2+offset(Follow.up)+Goodman.Scale,data = articles2,family="poisson"));
estim <- S$coefficients[2,1]
n <- dim(articles2)[1]; desv <- S$coefficients[2,2]; estim.ET <- exp(estim); se.ET <- desv
LL.estim.ET <- exp(estim-qt(0.975,dim(articles2)[1]-1)*desv); UL.estim.ET <- exp(estim+qt(0.975,dim(articles2)[1]-1)*desv)
# Unadjusted
S <- summary(m1 <- glm(NC ~ Group2,data = articles2,family="poisson"));
estim <- S$coefficients[2,1]
n <- dim(articles2)[1]; desv <-S$coefficients[2,2]; estim.ET.noadj <- exp(estim); se.ET.noadj <- desv
LL.estim.ET.noadj <- exp(estim-qt(0.975,dim(articles2)[1]-1)*desv); UL.estim.ET.noadj <- exp(estim+qt(0.975,dim(articles2)[1]-1)*desv)
# REPORTING GUIDELINES
articles2$Group3[articles2$Group=="R" | articles2$Group=="S"] <- "R+S"
articles2$Group3[articles2$Group=="C" | articles2$Group=="SC"] <- "C+SC"
articles2$Group3 <- as.factor(articles2$Group3)
articles2$Group3 <- relevel(articles2$Group3,ref="R+S")
# Adjusted
S <- summary(m1 <- glm(NC ~ as.factor(Group3)+offset(Follow.up)+Goodman.Scale,data = articles2,family="poisson"));
estim <- S$coefficients[2,1]
n <- dim(articles2)[1]; desv <-S$coefficients[2,2]; estim.PLOS.1 <- exp(estim); se.PLOS.1 <- desv
LL.estim.PLOS.1 <- exp(estim-qt(0.975,dim(articles2)[1]-1)*desv); UL.estim.PLOS.1 <- exp(estim+qt(0.975,dim(articles2)[1]-1)*desv)
# Unadjusted
S <- summary(m1 <- glm(NC ~ as.factor(Group3),data = articles2,family="poisson"));
estim <- S$coefficients[2,1]
n <- dim(articles2)[1]; desv <-S$coefficients[2,2]; estim.PLOS.1.noadj <- exp(estim); se.PLOS.1.noadj <- desv
LL.estim.PLOS.1.noadj <- exp(estim-qt(0.975,dim(articles2)[1]-1)*desv); UL.estim.PLOS.1.noadj <- exp(estim+qt(0.975,dim(articles2)[1]-1)*desv)
# GRAPHIC - Forest plot
poin <- c(0,15); lin <- c(1,1); wid <- c(1,2); pos <- c(2:1); etiq <- c("(n=49 vs 50)","(n=53 vs 46)")
win.graph(8,4); par(oma=c(0,8,0,7))
plot(NA, xlim = c(0.33,3), ylim=c(0,1.5),xaxt="n",yaxt="n", ylab="",xlab="",axes=F,log="x")
abline(v=1,lty=2); axis(1,c(1/3.5,0.33,0.5,1,2,3,3.5),labels=F)
mtext(c("1/3","1/2","1","2","3"),las=1,side=1,at=c(0.33,0.5,1,2,3),line=0.5)
# ET
segments(LL.estim.ET.noadj,1.2,UL.estim.ET.noadj,1.2,lwd=wid[1]); points(estim.ET.noadj,1.2,pch=poin[1],cex=0.7/sqrt(se.ET.noadj))
segments(LL.estim.ET,0.9,UL.estim.ET,0.9,lwd=wid[1]); points(estim.ET,0.9,pch=poin[2],cex=0.7/sqrt(se.ET))
# IQ
segments(LL.estim.PLOS.1.noadj,0.4,UL.estim.PLOS.1.noadj,0.4,lwd=wid[1]);points(estim.PLOS.1.noadj,0.4,pch=poin[1],cex=0.7/sqrt(se.PLOS.1.noadj))
segments(LL.estim.PLOS.1,0.1,UL.estim.PLOS.1,0.1,lwd=wid[1]);points(estim.PLOS.1,0.1,pch=poin[2],cex=0.7/sqrt(se.PLOS.1))
# Labels
mtext("Statistician",las=2,side=2,at=1,line=5.2,font=2,cex = 1.2)
mtext(etiq[1],las=2,side=2,at=0.8,line=5,cex = 1.2)
mtext("Reporting Guidelines",las=2,side=2,at=0.3,line=0.5,font=2,cex = 1.2)
mtext(etiq[2],las=2,side=2,at=0.1,line=5,cex = 1.2)
mtext("Mean ratio (CI 95%)",las=2,side=4,at=1.5,line=0.5,font=2)
mtext(paste("1.03","(","0.90",";","1.17",")"),side=4,las=2,at=1.2,line=1)
mtext(paste("1.00","(","0.88",";","1.14",")"),side=4,las=2,at=0.9,line=1)
mtext(paste("1.25","(","1.10",";","1.42",")"),side=4,las=2,at=0.4,line=1)
mtext(paste("1.28","(","1.13",";","1.46",")"),side=4,las=2,at=0.1,line=1)
mtext("Higher in intervention",las=2,side=2,at=-0.5,line=-21)
mtext("Higher in control",las=2,side=2,at=-0.5,line=-4)


################
##- Figure SM5.
################
##-- Citations-per-year mean ratio by Study type obtained from raw estimation.
##-- Point effect estimates and 95%CI are obtained through assumption-free resampling methods.

log.mean.ratio <- function(dat) {d=dat; m0=mean(d$response[d$group=="R"],na.rm=T); m1=mean(d$response[d$group=="SC"],na.rm=T); log(m1/m0)} # Function to find log(mean ratio)
# ET study
# Cross-sectional
response <- datos$NC[datos$Study=="ET" & datos$Study.type=="Cross-sectional"]/datos$Follow.up[datos$Study=="ET" & datos$Study.type=="Cross-sectional"]
group <- datos$Group[datos$Study=="ET" & datos$Study.type=="Cross-sectional"]; dat <- data.frame(response,group=as.factor(group)); estim <- c()
for (i in 1:dim(dat)[1]){  d <- dat[-i,];  estim[i] <- log.mean.ratio(d)}
estim[dim(dat)[1]+1] <- log.mean.ratio(dat); n <- dim(dat)[1]; desv <-sqrt(((n-1)/n)*sum((estim-mean(estim))^2)); estim.ET.cs <- round(exp(mean(estim)),2); se.ET.cs <- desv
LL.estim.ET.cs <- round(exp(mean(estim)-qt(0.975,dim(datos[datos$Study=="ET",])[1]-1)*desv),2); UL.estim.ET.cs <- round(exp(mean(estim)+qt(0.975,dim(datos[datos$Study=="ET",])[1]-1)*desv),2)
# Intervention
response <- datos$NC[datos$Study=="ET" & datos$Study.type=="Intervention"]/datos$Follow.up[datos$Study=="ET" & datos$Study.type=="Intervention"]
group <- datos$Group[datos$Study=="ET" & datos$Study.type=="Intervention"]; dat <- data.frame(response,group=as.factor(group)); estim <- c()
for (i in 1:dim(dat)[1]){  d <- dat[-i,];  estim[i] <- log.mean.ratio(d)}
estim[dim(dat)[1]+1] <- log.mean.ratio(dat); n <- dim(dat)[1]; desv <-sqrt(((n-1)/n)*sum((estim-mean(estim))^2)); estim.ET.i <- round(exp(mean(estim)),2); se.ET.i <- desv
LL.estim.ET.i <- round(exp(mean(estim)-qt(0.975,dim(datos[datos$Study=="ET",])[1]-1)*desv),2); UL.estim.ET.i <- round(exp(mean(estim)+qt(0.975,dim(datos[datos$Study=="ET",])[1]-1)*desv),2)
# Longitudinal
response <- datos$NC[datos$Study=="ET" & datos$Study.type=="Longitudinal"]/datos$Follow.up[datos$Study=="ET" & datos$Study.type=="Longitudinal"]
group <- datos$Group[datos$Study=="ET" & datos$Study.type=="Longitudinal"]; dat <- data.frame(response,group=as.factor(group)); estim <- c()
for (i in 1:dim(dat)[1]){  d <- dat[-i,];  estim[i] <- log.mean.ratio(d)}
estim[dim(dat)[1]+1] <- log.mean.ratio(dat); n <- dim(dat)[1]; desv <-sqrt(((n-1)/n)*sum((estim-mean(estim))^2)); estim.ET.l <- round(exp(mean(estim)),2); se.ET.l <- desv
LL.estim.ET.l <- round(exp(mean(estim)-qt(0.975,dim(datos[datos$Study=="ET",])[1]-1)*desv),2); UL.estim.ET.l <- round(exp(mean(estim)+qt(0.975,dim(datos[datos$Study=="ET",])[1]-1)*desv),2)
# Other
response <- datos$NC[datos$Study=="ET" & datos$Study.type=="Other"]/datos$Follow.up[datos$Study=="ET" & datos$Study.type=="Other"]
group <- datos$Group[datos$Study=="ET" & datos$Study.type=="Other"]; dat <- data.frame(response,group=as.factor(group)); estim <- c(); estim[1] <- log.mean.ratio(dat)
for (i in 1:dim(dat)[1]){  d <- dat[-i,];  estim[i] <- log.mean.ratio(d)}
n <- dim(dat)[1]; desv <-sqrt(((n-1)/n)*sum((estim-mean(estim))^2)); estim.ET.o <- round(exp(mean(estim)),2); se.ET.o <- desv
LL.estim.ET.o <- round(exp(mean(estim)-qt(0.975,dim(datos[datos$Study=="ET",])[1]-1)*desv),2); UL.estim.ET.o <- round(exp(mean(estim)+qt(0.975,dim(datos[datos$Study=="ET",])[1]-1)*desv),2)
ET <- c(estim.ET.cs, estim.ET.i, estim.ET.l, estim.ET.o)
ET.LL <- c(LL.estim.ET.cs, LL.estim.ET.i, LL.estim.ET.l, LL.estim.ET.o)
ET.UL <- c(UL.estim.ET.cs, UL.estim.ET.i, UL.estim.ET.l, UL.estim.ET.o)
se.ET <- c(se.ET.cs, se.ET.i, se.ET.l, se.ET.o)
# PLOS study: Both interventions effect (R vs SC)
# Cross-sectional
response <- datos$NC[datos$Study=="IQ" & (datos$Group=="R" | datos$Group=="SC") & datos$Study.type=="Cross-sectional"]/
  datos$Follow.up[datos$Study=="IQ" & (datos$Group=="R" | datos$Group=="SC") & datos$Study.type=="Cross-sectional"]
group <- datos$Group[datos$Study=="IQ" & (datos$Group=="R" | datos$Group=="SC") & datos$Study.type=="Cross-sectional"]
dat <- data.frame(response,group=as.factor(group)); estim <- c(); estim[1] <- log.mean.ratio(dat)
for (i in 2:dim(dat)[1]){  d <- dat[-i,];  estim[i] <- log.mean.ratio(d)}
n <- dim(dat)[1]; desv <-sqrt(((n-1)/n)*sum((estim-mean(estim,na.rm=T))^2)); estim.PLOS.cs <- round(exp(mean(estim,na.rm=T)),2); se.PLOS.cs <- desv
LL.estim.PLOS.cs <- round(exp(mean(estim,na.rm=T)-qt(0.975,dim(datos[datos$Study=="IQ" & (datos$Group=="R" | datos$Group=="SC"),])[1]-1)*desv),2)
UL.estim.PLOS.cs <- round(exp(mean(estim,na.rm=T)+qt(0.975,dim(datos[datos$Study=="IQ" & (datos$Group=="R" | datos$Group=="SC"),])[1]-1)*desv),2)
# Intervention
response <- datos$NC[datos$Study=="IQ" & (datos$Group=="R" | datos$Group=="SC") & datos$Study.type=="Intervention"]/
  datos$Follow.up[datos$Study=="IQ" & (datos$Group=="R" | datos$Group=="SC") & datos$Study.type=="Intervention"]
group <- datos$Group[datos$Study=="IQ" & (datos$Group=="R" | datos$Group=="SC") & datos$Study.type=="Intervention"]
dat <- data.frame(response,group=as.factor(group)); estim <- c(); estim[1] <- log.mean.ratio(dat)
for (i in 1:dim(dat)[1]){  d <- dat[-i,];  estim[i] <- log.mean.ratio(d)}
n <- dim(dat)[1]; desv <-sqrt(((n-1)/n)*sum((estim-mean(estim,na.rm=T))^2)); estim.PLOS.i <- round(exp(mean(estim,na.rm=T)),2); se.PLOS.i <- desv
LL.estim.PLOS.i <- round(exp(mean(estim,na.rm=T)-qt(0.975,dim(datos[datos$Study=="IQ" & (datos$Group=="R" | datos$Group=="SC"),])[1]-1)*desv),2)
UL.estim.PLOS.i <- round(exp(mean(estim,na.rm=T)+qt(0.975,dim(datos[datos$Study=="IQ" & (datos$Group=="R" | datos$Group=="SC"),])[1]-1)*desv),2)
# Longitudinal
response <- datos$NC[datos$Study=="IQ" & (datos$Group=="R" | datos$Group=="SC") & datos$Study.type=="Longitudinal"]/
  datos$Follow.up[datos$Study=="IQ" & (datos$Group=="R" | datos$Group=="SC") & datos$Study.type=="Longitudinal"]
group <- datos$Group[datos$Study=="IQ" & (datos$Group=="R" | datos$Group=="SC") & datos$Study.type=="Longitudinal"]
dat <- data.frame(response,group=as.factor(group)); estim <- c(); estim[1] <- log.mean.ratio(dat)
for (i in 1:dim(dat)[1]){  d <- dat[-i,];  estim[i] <- log.mean.ratio(d)}
n <- dim(dat)[1]; desv <-sqrt(((n-1)/n)*sum((estim-mean(estim,na.rm=T))^2))
estim.PLOS.l <- round(exp(mean(estim,na.rm=T)),2); se.PLOS.l <- desv
LL.estim.PLOS.l <- round(exp(mean(estim,na.rm=T)-qt(0.975,dim(datos[datos$Study=="IQ" & (datos$Group=="R" | datos$Group=="SC"),])[1]-1)*desv),2)
UL.estim.PLOS.l <- round(exp(mean(estim,na.rm=T)+qt(0.975,dim(datos[datos$Study=="IQ" & (datos$Group=="R" | datos$Group=="SC"),])[1]-1)*desv),2)
# Other
response <- datos$NC[datos$Study=="IQ" & (datos$Group=="R" | datos$Group=="SC") & datos$Study.type=="Other"]/
  datos$Follow.up[datos$Study=="IQ" & (datos$Group=="R" | datos$Group=="SC") & datos$Study.type=="Other"]
group <- datos$Group[datos$Study=="IQ" & (datos$Group=="R" | datos$Group=="SC") & datos$Study.type=="Other"]
dat <- data.frame(response,group=as.factor(group)); estim <- c(); estim[1] <- log.mean.ratio(dat)
for (i in 1:dim(dat)[1]){  d <- dat[-i,];  estim[i] <- log.mean.ratio(d)}
n <- dim(dat)[1]; desv <-sqrt(((n-1)/n)*sum((estim-mean(estim,na.rm=T))^2)); estim.PLOS.o <- round(exp(mean(estim,na.rm=T)),2); se.PLOS.o <- desv
LL.estim.PLOS.o <- round(exp(mean(estim,na.rm=T)-qt(0.975,dim(datos[datos$Study=="IQ" & (datos$Group=="R" | datos$Group=="SC"),])[1]-1)*desv),2)
UL.estim.PLOS.o <- round(exp(mean(estim,na.rm=T)+qt(0.975,dim(datos[datos$Study=="IQ" & (datos$Group=="R" | datos$Group=="SC"),])[1]-1)*desv),2)
IQ <- c(estim.PLOS.cs, estim.PLOS.i, estim.PLOS.l, estim.PLOS.o)
IQ.LL <- c(LL.estim.PLOS.cs, LL.estim.PLOS.i, LL.estim.PLOS.l, LL.estim.PLOS.o)
IQ.UL <- c(UL.estim.PLOS.cs, UL.estim.PLOS.i, UL.estim.PLOS.l, UL.estim.PLOS.o)
se.IQ <- c(se.PLOS.cs, se.PLOS.i, se.PLOS.l, se.PLOS.o)
# Meta-analysis
logs.cs <- c(log(ET[1]),log(IQ[1])); selogs.cs <- c(se.ET[1],se.IQ[1])
names <- c("ET study", "IQ study")
b <- data.frame(names,logs.cs,selogs.cs)
d <- meta.summaries(b$logs.cs, b$selogs.cs, names=b$names, method="fix", logscale=TRUE)
estim.meta.cs <- round(exp(d$summary),2)
se.estim.meta.cs <- d$se.summary
LL.estim.meta.cs <- round(summary(d)$summci[1],2)
UL.estim.meta.cs <- round(summary(d)$summci[3],2)
logs.i <- c(log(ET[2]),log(IQ[2])); selogs.i <- c(se.ET[2],se.IQ[2])
names <- c("ET study", "IQ study")
b <- data.frame(names,logs.i,selogs.i)
d <- meta.summaries(b$logs.i, b$selogs.i, names=b$names, method="fix", logscale=TRUE)
estim.meta.i <- round(exp(d$summary),2)
se.estim.meta.i <- d$se.summary
LL.estim.meta.i <- round(summary(d)$summci[1],2)
UL.estim.meta.i <- round(summary(d)$summci[3],2)
logs.l <- c(log(ET[3]),log(IQ[3])); selogs.l <- c(se.ET[3],se.IQ[3])
names <- c("ET study", "IQ study")
b <- data.frame(names,logs.l,selogs.l)
d <- meta.summaries(b$logs.l, b$selogs.l, names=b$names, method="fix", logscale=TRUE)
estim.meta.l <- round(exp(d$summary),2)
se.estim.meta.l <- d$se.summary
LL.estim.meta.l <- round(summary(d)$summci[1],2)
UL.estim.meta.l <- round(summary(d)$summci[3],2)
logs.o <- c(log(ET[4]),log(IQ[4])); selogs.o <- c(se.ET[4],se.IQ[4])
names <- c("ET study", "IQ study")
b <- data.frame(names,logs.o,selogs.o)
d <- meta.summaries(b$logs.o, b$selogs.o, names=b$names, method="fix", logscale=TRUE)
estim.meta.o <- round(exp(d$summary),2)
se.estim.meta.o <- d$se.summary
LL.estim.meta.o <- round(summary(d)$summci[1],2)
UL.estim.meta.o <- round(summary(d)$summci[3],2)
estim.meta <- c(estim.meta.cs,estim.meta.i,estim.meta.l,estim.meta.o)
se.estim.meta <- c(se.estim.meta.cs,se.estim.meta.i,se.estim.meta.l,se.estim.meta.o)
LL.estim.meta <- c(LL.estim.meta.cs, LL.estim.meta.i, LL.estim.meta.l, LL.estim.meta.o)
UL.estim.meta <- c(UL.estim.meta.cs, UL.estim.meta.i, UL.estim.meta.l, UL.estim.meta.o)
# GRAPHIC Forest plot
poin <- c(0,15); lin <- c(1,1); wid <- c(1,2); pos <- c(2:1); etiq <- c("(n=9 vs 17)","(n=8 vs 8)", "(n=20 vs 18)", "(n=4 vs 8)","(n=7 vs 3)","(n=3 vs 7)", "(n=12 vs 10)", "(n=5 vs 4)")
win.graph(8,8); par(oma=c(0,8,0,7))
plot(NA, xlim = c(1/9,9), ylim=c(0,13),xaxt="n",yaxt="n", ylab="",xlab="",axes=F,log="x")
abline(v=1,lty=2); axis(1,c(1/11,1/10,1/8,1/6,1/4,1/2,1,2,4,6,8,10,11),labels=F)
mtext(c("1/10","1/8","1/6","1/4","1/2","1","2","4","6","8","10"),las=1,side=1,at=c(1/10,1/8,1/6,1/4,1/2,1,2,4,6,8,10),line=0.5,cex=0.6)
# Meta cross-sectional
polygon(x=c(LL.estim.meta.cs,estim.meta.cs,UL.estim.meta.cs),y=c(13.1,13.1+(0.15/(sqrt(se.estim.meta[1])*(log(UL.estim.meta.cs)-log(LL.estim.meta.cs)))),13.1))
polygon(x=c(LL.estim.meta.cs,estim.meta.cs,UL.estim.meta.cs),y=c(13.1,13.1-(0.15/(sqrt(se.estim.meta[1])*(log(UL.estim.meta.cs)-log(LL.estim.meta.cs)))),13.1))
# Meta interventional
polygon(x=c(LL.estim.meta.i,estim.meta.i,UL.estim.meta.i),y=c(12.1,12.1+(0.15/(sqrt(se.estim.meta[2])*(log(UL.estim.meta.i)-log(LL.estim.meta.i)))),12.1))
polygon(x=c(LL.estim.meta.i,estim.meta.i,UL.estim.meta.i),y=c(12.1,12.1-(0.15/(sqrt(se.estim.meta[2])*(log(UL.estim.meta.i)-log(LL.estim.meta.i)))),12.1))
# Meta longitudinal
polygon(x=c(LL.estim.meta.l,estim.meta.l,UL.estim.meta.l),y=c(11.1,11.1+(0.15/(sqrt(se.estim.meta[3])*(log(UL.estim.meta.l)-log(LL.estim.meta.l)))),11.1))
polygon(x=c(LL.estim.meta.l,estim.meta.l,UL.estim.meta.l),y=c(11.1,11.1-(0.15/(sqrt(se.estim.meta[3])*(log(UL.estim.meta.l)-log(LL.estim.meta.l)))),11.1))
# Meta other
polygon(x=c(LL.estim.meta.o,estim.meta.o,UL.estim.meta.o),y=c(10.1,10.1+(0.15/(sqrt(se.estim.meta[4])*(log(UL.estim.meta.o)-log(LL.estim.meta.o)))),10.1))
polygon(x=c(LL.estim.meta.o,estim.meta.o,UL.estim.meta.o),y=c(10.1,10.1-(0.15/(sqrt(se.estim.meta[4])*(log(UL.estim.meta.o)-log(LL.estim.meta.o)))),10.1))
# ET cross-sectional
segments(ET.LL[1],8.1,ET.UL[1],8.1,lwd=wid[1])
points(ET[1],8.1,pch=poin[1],cex=1.5/sqrt(se.ET[1]))
# ET intervention
segments(ET.LL[2],7.1,ET.UL[2],7.1,lwd=wid[1])
points(ET[2],7.1,pch=poin[1],cex=1.5/sqrt(se.ET[2]))
# ET longitudinal
segments(ET.LL[3],6.1,ET.UL[3],6.1,lwd=wid[1])
points(ET[3],6.1,pch=poin[1],cex=1.5/sqrt(se.ET[3]))
# ET other
segments(ET.LL[4],5.1,ET.UL[4],5.1,lwd=wid[1])
points(ET[4],5.1,pch=poin[1],cex=1.5/sqrt(se.ET[4]))
# IQ cross-sectional
arrows(IQ.LL[1],3.1,10,3.1,length=0.1,lwd=wid[1]); points(IQ[1],3.1,pch=poin[1],cex=1.5/sqrt(se.IQ[1]))
# IQ intervention
segments(IQ.LL[2],2.1,IQ.UL[2],2.1,lwd=wid[1])
points(IQ[2],2.1,pch=poin[1],cex=1.5/sqrt(se.IQ[2]))
# IQ longitudinal
segments(IQ.LL[3],1.1,IQ.UL[3],1.1,lwd=wid[1])
points(IQ[3],1.1,pch=poin[1],cex=1.5/sqrt(se.IQ[3]))
# IQ other
arrows(IQ.LL[4],0.1,10,0.1,length=0.1,lwd=wid[1]);
points(IQ[4],0.1,pch=poin[1],cex=1.5/sqrt(se.IQ[4]))
# Labels
mtext("Join MA",las=2,side=2,at=14.5,line=7,font=2,cex = 1.2)
mtext("estimated effect",las=2,side=2,at=14,line=3.5,font=2,cex = 1.2)
mtext("Cross-sectional",las=2,side=2,at=13.1,line=2.5,font=2,cex=1)
mtext("Interventional",las=2,side=2,at=12.1,line=3.3,font=2,cex=1)
mtext("Longitudinal",las=2,side=2,at=11.1,line=3.7,font=2,cex=1)
mtext("Other",las=2,side=2,at=10.1,line=6.5,font=2,cex=1)
mtext("ET study",las=2,side=2,at=8.8,line=6.5,font=2,cex = 1.2)
mtext("Cross-sectional",las=2,side=2,at=8.1,line=2.5,font=2,cex=1); mtext(etiq[1],las=2,side=2,at=7.7,line=4.7)
mtext("Interventional",las=2,side=2,at=7.1,line=3.3,font=2,cex=1); mtext(etiq[2],las=2,side=2,at=6.7,line=5.2)
mtext("Longitudinal",las=2,side=2,at=6.1,line=3.7,font=2,cex=1); mtext(etiq[3],las=2,side=2,at=5.7,line=4.3)
mtext("Other",las=2,side=2,at=5.1,line=6.5,font=2,cex=1); mtext(etiq[4],las=2,side=2,at=4.7,line=4.7)
mtext("IQ study",las=2,side=2,at=3.8,line=6.5,font=2,cex = 1.2)
mtext("Cross-sectional",las=2,side=2,at=3.1,line=2.5,font=2,cex=1); mtext(etiq[5],las=2,side=2,at=2.7,line=5.2)
mtext("Interventional",las=2,side=2,at=2.1,line=3.3,font=2,cex=1); mtext(etiq[6],las=2,side=2,at=1.7,line=5.2)
mtext("Longitudinal",las=2,side=2,at=1.1,line=3.7,font=2,cex=1); mtext(etiq[7],las=2,side=2,at=0.7,line=4.3)
mtext("Other",las=2,side=2,at=0.1,line=6.5,font=2,cex=1); mtext(etiq[8],las=2,side=2,at=-0.3,line=5.2)
mtext("Mean ratio (CI 95%)",las=2,side=4,at=14.2,line=0.5,font=2)
mtext(paste(estim.meta,"(",LL.estim.meta,";",UL.estim.meta,")"),side=4,las=2,at=c(13.1, 12.1,11.1,10.1),line=1)
mtext(paste(ET,"(",ET.LL,";",ET.UL,")"),side=4,las=2,at=c(8.1, 7.1,6.1,5.1),line=1)
mtext(paste(IQ,"(",IQ.LL,";",IQ.UL,")"),side=4,las=2,at=c(3.1, 2.1,1.1,0.1),line=1)
mtext("Higher in intervention",las=2,side=2,at=-1.3,line=-20,cex=0.8)
mtext("Higher in control",las=2,side=2,at=-1.3,line=-3.5,cex=0.8)


#####################################
##- Figure SM6. Sensitivity analysis
#####################################
##-- Citations-per-year mean ratio obtained from other possible models ().
##-- Point effect estimates and 95%CI are obtained through assumption-free resampling methods.

library(pscl)
#- ET: R vs SC
# Q-P Adjusted
S <- summary(model.quasiPois <- glm(NC ~ as.factor(Group)+offset(Follow.up)+Goodman.Scale, data = datos[datos$Study=="ET",] ,family="quasipoisson"))
estim <- c(); estim_theta <- c(); estim[1] <- S$coefficients[2,1]
for (i in 1:dim(datos[datos$Study=="ET",])[1]){d <- datos[datos$Study=="ET",][-i,]
S <- summary(m1 <- glm(NC ~ as.factor(Group)+offset(Follow.up)+Goodman.Scale, data = d,family="quasipoisson"))
estim[i] <- S$coefficients[2,1]; estim_theta[i] <- S$coefficients[2,2]}
n <- dim(datos[datos$Study=="ET",])[1]; desv <- mean(estim_theta)
ET_OR1 <- exp(mean(estim)); ET_OR1_inf <- exp(mean(estim)-qt(0.975,dim(datos[datos$Study=="ET",])[1]-1)*desv)
ET_OR1_sup <- exp(mean(estim)+qt(0.975,dim(datos[datos$Study=="ET",])[1]-1)*desv)
ET_OR1.qpois <- ET_OR1; se.Adj.ET.qpois <- desv
d1_o_qp <- data.frame(x=c("C vs CSK"),y=round(c(ET_OR1),2), ylo=round(c(ET_OR1_inf),2),yhi=round(c(ET_OR1_sup),2))
# Adjusted NB (offset(FU) + Baseline Goodman scale)
library(MASS)
S <- summary(m1 <- glm.nb(NC ~ as.factor(Group)+offset(Follow.up)+Goodman.Scale, data = datos[datos$Study=="ET",]))
estim <- c(); estim[1] <- S$coefficients[2,1]
for (i in 1:dim(datos[datos$Study=="ET",])[1]){d <- datos[datos$Study=="ET",][-i,]
S <- summary(m1 <- glm.nb(NC ~ as.factor(Group)+offset(Follow.up)+Goodman.Scale, data = d)); estim[i] <- S$coefficients[2,1]}
n <- dim(datos[datos$Study=="ET",])[1]
desv <-sqrt(((n-1)/n)*sum((estim-mean(estim))^2)); ET_OR1 <- exp(mean(estim))
ET_OR1_inf <- exp(mean(estim)-qt(0.975,dim(datos[datos$Study=="ET",])[1]-1)*desv); ET_OR1_sup <- exp(mean(estim)+qt(0.975,dim(datos[datos$Study=="ET",])[1]-1)*desv)
d2_o <- data.frame(x=c("C vs CSK"),y=round(c(ET_OR1),2), ylo=round(c(ET_OR1_inf),2),yhi=round(c(ET_OR1_sup),2))
ET_OR1.nb <- ET_OR1; se.Adj.ET.nb <- desv
# Adjusted Zero-inflated NB model (offset(FU) + Baseline Goodman scale)
library(MASS)
S <- summary(m1 <- zeroinfl(NC ~ as.factor(Group)+offset(Follow.up)+Goodman.Scale, data = datos[datos$Study=="ET",], dist="negbin"))
estim <- c(); estim[1] <- S$coefficients$count[2,1]
for (i in 1:dim(datos[datos$Study=="ET",])[1]){d <- datos[datos$Study=="ET",][-i,]
S <- summary(m1 <- zeroinfl(NC ~ as.factor(Group)+offset(Follow.up)+Goodman.Scale, data = d, dist="negbin"))
estim[i] <- S$coefficients$count[2,1]}
n <- dim(datos[datos$Study=="ET",])[1]; desv <-sqrt(((n-1)/n)*sum((estim-mean(estim))^2)); ET_OR1 <- exp(mean(estim))
ET_OR1_inf <- exp(mean(estim)-qt(0.975,dim(datos[datos$Study=="ET",])[1]-1)*desv); ET_OR1_sup <- exp(mean(estim)+qt(0.975,dim(datos[datos$Study=="ET",])[1]-1)*desv)
d3_o <- data.frame(x=c("C vs CSK"),y=round(c(ET_OR1),2), ylo=round(c(ET_OR1_inf),2),yhi=round(c(ET_OR1_sup),2))
ET_OR1.zinb <- ET_OR1; se.Adj.ET.zinb <- desv
# PLOS: R vs SC
articles1 <- subset(datos[datos$Study=="IQ",],Group=="R" | Group=="SC")
# Adjusted Quasi-Poisson
S <- summary(model.quasiPois <- glm(NC ~ as.factor(Group)+offset(Follow.up)+Goodman.Scale, data = articles1 ,family="quasipoisson"))
estim <- c(); estim_theta <- c(); estim[1] <- S$coefficients[2,1]
for (i in 1:dim(articles1)[1]){d <- articles1[-i,]
S <- summary(m1 <- glm(NC ~ as.factor(Group)+offset(Follow.up)+Goodman.Scale, data = d,family="quasipoisson"))
estim[i] <- S$coefficients[2,1]; estim_theta[i] <- S$coefficients[2,2]}
n <- dim(articles1)[1]; desv <- mean(estim_theta)
PLOS_C_CSK_OR1 <- exp(mean(estim))
PLOS_C_CSK_OR1_inf <- exp(mean(estim)-qt(0.975,dim(articles1)[1]-1)*desv); PLOS_C_CSK_OR1_sup <- exp(mean(estim)+qt(0.975,dim(articles1)[1]-1)*desv)
d4_o_qp <- data.frame(x=c("C vs CSK"),y=round(c(PLOS_C_CSK_OR1),2), ylo=round(c(PLOS_C_CSK_OR1_inf),2),yhi=round(c(PLOS_C_CSK_OR1_sup),2))
PLOS_C_CSK_OR1.qpois <- PLOS_C_CSK_OR1; se.Adj.PLOS1.qpois <- desv
# Adjusted NB (offset(FU) + Baseline Goodman scale)
library(MASS)
S <- summary(m1 <- glm.nb(NC ~ as.factor(Group)+offset(Follow.up)+Goodman.Scale, data = articles1))
estim <- c(); estim[1] <- S$coefficients[2,1]
for (i in 1:dim(articles1)[1]){d <- articles1[-i,]
S <- summary(m1 <- glm.nb(NC ~ as.factor(Group)+offset(Follow.up)+Goodman.Scale, data = d)); estim[i] <- S$coefficients[2,1]}
n <- dim(articles1)[1]; desv <-sqrt(((n-1)/n)*sum((estim-mean(estim))^2)); PLOS_C_CSK_OR1 <- exp(mean(estim))
PLOS_C_CSK_OR1_inf <- exp(mean(estim)-qt(0.975,dim(articles1)[1]-1)*desv); PLOS_C_CSK_OR1_sup <- exp(mean(estim)+qt(0.975,dim(articles1)[1]-1)*desv)
d5_o <- data.frame(x=c("C vs CSK"),y=round(c(PLOS_C_CSK_OR1),2),ylo=round(c(PLOS_C_CSK_OR1_inf),2),yhi=round(c(PLOS_C_CSK_OR1_sup),2))
PLOS_C_CSK_OR1.nb <- PLOS_C_CSK_OR1; se.Adj.PLOS1.nb <- desv
# Adjusted Zero-inflated NB model (offset(FU) + Baseline Goodman scale)
library(MASS)
S <- summary(m1 <- zeroinfl(NC ~ as.factor(Group)+offset(Follow.up)+Goodman.Scale, data = articles1,dist="negbin"))
estim <- c(); estim[1] <- S$coefficients$count[2,1]
for (i in 1:dim(articles1)[1]){d <- articles1[-i,]
S <- summary(m1 <- zeroinfl(NC ~ as.factor(Group)+offset(Follow.up)+Goodman.Scale, data = d,dist="negbin")); estim[i] <- S$coefficients$count[2,1]}
n <- dim(articles1)[1]; desv <-sqrt(((n-1)/n)*sum((estim-mean(estim))^2)); PLOS_C_CSK_OR1 <- exp(mean(estim))
PLOS_C_CSK_OR1_inf <- exp(mean(estim)-qt(0.975,dim(articles1)[1]-1)*desv); PLOS_C_CSK_OR1_sup <- exp(mean(estim)+qt(0.975,dim(articles1)[1]-1)*desv)
d6_o <- data.frame(x=c("C vs CSK"),y=round(c(PLOS_C_CSK_OR1),2),ylo=round(c(PLOS_C_CSK_OR1_inf),2),yhi=round(c(PLOS_C_CSK_OR1_sup),2))
PLOS_C_CSK_OR1.zinb <- PLOS_C_CSK_OR1; se.Adj.PLOS1.zinb <- desv
# META-ANÁLISIS
library(rmeta)
# Quasi-Poisson
logs <- c(log(ET_OR1.qpois),log(PLOS_C_CSK_OR1.qpois)); selogs <- c(se.Adj.ET.qpois,se.Adj.PLOS1.qpois)
names <- c("ET study", "IQ study"); b <- data.frame(names,logs,selogs)
d <- meta.summaries(b$logs, b$selogs, names=b$names, method="fix", logscale=TRUE)
estim.meta.qpois <- round(exp(d$summary),2); LL.estim.meta.qpois <- round(exp(d$summary-1.96*d$se.summary),2)
UL.estim.meta.qpois <- round(exp(d$summary+1.96*d$se.summary),2); se.estim.meta.qpois <- d$se.summary
# NB
logs <- c(log(ET_OR1.nb),log(PLOS_C_CSK_OR1.nb)); selogs <- c(se.Adj.ET.nb,se.Adj.PLOS1.nb)
names <- c("ET study", "IQ study"); b <- data.frame(names,logs,selogs)
d <- meta.summaries(b$logs, b$selogs, names=b$names,method="fix", logscale=TRUE)
estim.meta.nb <- round(exp(d$summary),2); LL.estim.meta.nb <- round(exp(d$summary-1.96*d$se.summary),2)
UL.estim.meta.nb <- round(exp(d$summary+1.96*d$se.summary),2); se.estim.meta.nb <- d$se.summary
# ZINB
logs <- c(log(ET_OR1.zinb),log(PLOS_C_CSK_OR1.zinb)); selogs <- c(se.Adj.ET.zinb,se.Adj.PLOS1.zinb)
names <- c("ET study", "IQ study"); b <- data.frame(names,logs,selogs)
d <- meta.summaries(b$logs, b$selogs, names=b$names,method="fix", logscale=TRUE)
estim.meta.zinb <- round(exp(d$summary),2); LL.estim.meta.zinb <- round(exp(d$summary-1.96*d$se.summary),2)
UL.estim.meta.zinb <- round(exp(d$summary+1.96*d$se.summary),2); se.estim.meta.zinb <- d$se.summary
# GRAPHIC - FOREST PLOT
ET <- rbind(d1_o_qp,d2_o,d3_o); PLOS_CvsCSK <- rbind(d4_o_qp,d5_o,d6_o)
poin <- c(15,0); lin <- c(1,2,3,4); wid <- c(2,2); cols <- c("black","gray44","gray64","grey")
etiq <- c("(n=41 vs 51)", "(n=27 vs 24)")
win.graph(14,12); par(oma=c(0,8,0,7))
plot(NA, xlim = c(0.33,3), ylim=c(0,6.5),xaxt="n",yaxt="n", ylab="",xlab="",axes=F,log="x")
abline(v=1,lty=2); axis(1,c(0.33,0.5,1,2,3),labels=F)
mtext(c("1/3","1/2","1","2","3"),las=1,side=1,at=c(0.33,0.5,1,2,3),line=0.5)
par(xpd=NA)
legend(0.5,7.8,horiz=F,bty="n",c("Adjusted Quasi-Poisson","Adjusted Negative Binomial",
                                 "Adjusted Zero-Inflated Negative Binomial"),lty=lin,lwd=wid,pch=15,col=cols)
par(xpd=FALSE)
# Pooled estimation (Meta-analisis)
segments(LL.estim.meta.qpois,5.5,UL.estim.meta.qpois,5.5,lwd=wid[1],lty=lin[1],col=cols[1]); points(estim.meta.qpois,5.5,pch=poin[1],cex=1/se.estim.meta.qpois*0.6,col=cols[1])
segments(LL.estim.meta.nb,5.1,UL.estim.meta.nb,5.1,lwd=wid[1],lty=lin[2],col=cols[2]); points(estim.meta.nb,5.1,pch=poin[1],cex=1/se.estim.meta.nb*0.6,col=cols[2])
segments(LL.estim.meta.nb,4.7,UL.estim.meta.zinb,4.7,lwd=wid[1],lty=lin[3],col=cols[3]); points(estim.meta.zinb,4.7,pch=poin[1],cex=1/se.estim.meta.zinb*0.6,col=cols[3])
# ET
segments(ET$ylo[1],3.5,ET$yhi[1],3.5,lwd=wid[1],lty=lin[1],col=cols[1]); points(ET$y[1],3.5,pch=poin[1],cex=1.1/se.Adj.ET.qpois*0.6,col=cols[1])
segments(ET$ylo[2],3.1,ET$yhi[2],3.1,lwd=wid[2],lty=lin[2],col=cols[2]); points(ET$y[2],3.1,pch=poin[1],cex=1.1/se.Adj.ET.nb*0.6,col=cols[2])
segments(ET$ylo[3],2.7,ET$yhi[3],2.7,lwd=wid[1],lty=lin[3],col=cols[3]); points(ET$y[3],2.7,pch=poin[1],cex=1.1/se.Adj.ET.zinb*0.6,col=cols[3])
# IQ
segments(PLOS_CvsCSK$ylo[1],1.5,PLOS_CvsCSK$yhi[1],1.5,lwd=wid[1],lty=lin[1],col=cols[1])
points(PLOS_CvsCSK$y[1],1.5,pch=poin[1],cex=1.1/se.Adj.PLOS1.qpois*0.6,col=cols[1])
segments(PLOS_CvsCSK$ylo[2],1.1,PLOS_CvsCSK$yhi[2],1.1,lwd=wid[2],lty=lin[2],col=cols[2])
points(PLOS_CvsCSK$y[2],1.1,pch=poin[1],cex=1.1/se.Adj.PLOS1.nb*0.6,col=cols[2])
segments(PLOS_CvsCSK$ylo[3],0.7,PLOS_CvsCSK$yhi[3],0.7,lwd=wid[1],lty=lin[3],col=cols[3])
points(PLOS_CvsCSK$y[3],0.7,pch=poin[1],cex=1.1/se.Adj.PLOS1.zinb*0.6,col=cols[3])
# Labels
mtext("ET study",las=2,side=2,at=3.3,line=2,font=2,cex=1.2); mtext(etiq[1],las=2,side=2,at=3,line=2)
mtext("IQ study",las=2,side=2,at=1.3,line=2,font=2,cex=1.2); mtext(etiq[2],las=2,side=2,at=1,line=1.8)
mtext("Mean ratio (CI 95%)",las=2,side=4,at=6,line=0.6,font=2,cex=1)
mtext(paste(ET$y[c(1,2,3)],"(",ET$ylo[c(1,2,3)],";",ET$yhi[c(1,2,3)],")"),side=4,las=2,at=c(3.5,3.1,2.7),line=1.5,cex=0.9)
mtext(paste(PLOS_CvsCSK$y[c(1,2,3)],"(",PLOS_CvsCSK$ylo[c(1,2,3)],";",PLOS_CvsCSK$yhi[c(1,2,3)],")"),side=4,las=2,at=c(1.5,1.1,0.7),line=1.5,cex=0.9)
mtext(paste(estim.meta.qpois,"(",LL.estim.meta.qpois,";",UL.estim.meta.qpois,")"),side=4,las=2,at=5.5,line=1.5,cex=0.9)
mtext(paste(estim.meta.nb,"(",LL.estim.meta.nb,";",UL.estim.meta.nb,")"),side=4,las=2,at=5.1,line=1.5,cex=0.9)
mtext(paste(estim.meta.zinb,"(",LL.estim.meta.zinb,";",UL.estim.meta.zinb,")"),side=4,las=2,at=4.7,line=1.5,cex=0.9)
mtext("Join MA",las=2,side=2,at=5.3,line=2,font=2,cex=1.2)
mtext("estimated effect",las=2,side=2,at=5.1,line=-1.8,font=2,cex=1.2)
mtext("Higher in intervention",las=1,side=1,at=3,line=2)
mtext("Higher in control",las=1,side=1,at=1/3,line=2)


################
##- Figure SM7.
################
##-- Cumulative Distribution Functions (CDFs) for Zero-Inflated Poisson, Poisson, Quasi-Poisson, Negative Binomial
##-- and Zero-Inflated Negative Binomial models to compare the adjustment of these different models. ET study.

library(pscl)
library(boot)
library(fitdistrplus)    # fits distributions using maximum likelihood
library(gamlss)          # defines pdf, cdf of ZIP
#- ET STUDY
# Zero-Inflated Poisson
S <- summary(m1 <- zeroinfl(NC ~ 1, data = datos[datos$Study=="ET",])); mu_C = S$coefficients$count[1]; zero_C = S$coefficients$zero[1]
fit_zip = fitdist(datos$NC[datos$Study=="ET"], 'ZIP', start = list(mu = mu_C, sigma = zero_C),lower=c(-Inf, 0.001),upper=c(Inf, 1), optim.method="L-BFGS-B")
cdfcomp(fit_zip,lwd=2,legendtext="ZIP")
# Poisson
fit.pois <- fitdist(datos$NC[datos$Study=="ET"], 'pois')
# Quasi-Poisson
set.seed(1234); rqpois = function(n, lambda, phi){mu = lambda; k = mu/(phi - 1); m = rnbinom(n, mu = mu, size = k); return(m)}
n <- dim(datos[datos$Study=="ET",])[1]; lmd <- mean(datos$NC[datos$Study=="ET"])
model.quasiPois <- glm(NC ~ 1, data = datos[datos$Study=="ET",] ,family="quasipoisson")
disp <- summary(model.quasiPois)$dispersion; fit.qpois <- rqpois(n,lmd,phi=disp)
# Negative Binomial
fit.nb <- fitdist(datos$NC[datos$Study=="ET"], 'nbinom')
# Zero-Inflated Negative Binomial
model.zinb <- zeroinfl(NC ~ 1, data = datos[datos$Study=="ET",],dist="negbin")
S <- summary(model.zinb); k <- S$theta; lambda <- exp(S$coefficients$count[1]); omega = inv.logit(S$coefficients$zero[1])
set.seed(567); fit.zinb <- rZINBI(n, k,lambda, omega)
# Plot CDFs for ET study
windows()
cdfcomp(list(fit.nb,fit_zip, fit.pois),lwd=2,addlegend = F, fitlty=1, main="Empirical and theoretical CDFs for ET study")
lines(ecdf(fit.qpois),verticals=T,lwd=2,col="gold2",lty=1,pch=NA)
lines(ecdf(fit.zinb),verticals=T,lwd=2,col="cyan2",lty=1,pch=NA)
legend("bottomright",c("Empirical CFD","Zero-Inflated Poisson","Poisson","Quasi-Poisson","Negative Binomial","Zero-Inflated Negative Binomial")
       ,col=c("black","green","blue","gold2","red","cyan2"),lty=1,lwd=2)


################
##- Figure SM8.
################
##-- Cumulative Distribution Functions (CDFs) for Zero-Inflated Poisson, Poisson, Quasi-Poisson, Negative Binomial
##-- and Zero-Inflated Negative Binomial models to compare the adjustment of these different models. IQ study.

library(pscl)
library(boot)
library(fitdistrplus)    # fits distributions using maximum likelihood
library(gamlss)          # defines pdf, cdf of ZIP
#- IQ STUDY
# Zero-Inflated Poisson
S <- summary(m1 <- zeroinfl(NC ~ 1, data = datos[datos$Study=="IQ",])); mu_C = S$coefficients$count[1]; zero_C = S$coefficients$zero[2]
fit_zip = fitdist(datos$NC[datos$Study=="IQ"], 'ZIP', start = list(mu = mu_C, sigma = zero_C),lower=c(-Inf, 0.00001),upper=c(Inf, 1), optim.method="L-BFGS-B")
cdfcomp(fit_zip,lwd=2,legendtext="ZIP")
# Poisson
fit.pois <- fitdist(datos$NC[datos$Study=="IQ"], 'pois')
# Quasi-Poisson
set.seed(1234); rqpois = function(n, lambda, phi){mu = lambda; k = mu/(phi - 1); m = rnbinom(n, mu = mu, size = k); return(m)}
n <- dim(datos[datos$Study=="IQ",])[1]; lmd <- mean(datos$NC[datos$Study=="IQ"])
model.quasiPois <- glm(NC ~ 1, data = datos[datos$Study=="IQ",] ,family="quasipoisson")
disp <- summary(model.quasiPois)$dispersion; fit.qpois <- rqpois(n,lmd,phi=disp)
# Negative Binomial
fit.nb <- fitdist(datos$NC[datos$Study=="IQ"], 'nbinom')
# Zero-Inflated Negative Binomial
model.zinb <- zeroinfl(NC ~ 1, data = datos[datos$Study=="IQ",],dist="negbin")
S <- summary(model.zinb); k <- S$theta; lambda <- exp(S$coefficients$count[1]); omega = inv.logit(S$coefficients$zero[1])
set.seed(567); fit.zinb <- rZINBI(n, k,lambda, omega)
# Plot CDFs for IQ study
windows()
cdfcomp(list(fit.nb,fit_zip, fit.pois),lwd=2,addlegend = F, fitlty=1, main="Empirical and theoretical CDFs for IQ study")
lines(ecdf(fit.qpois),verticals=T,lwd=2,col="gold2",lty=1,pch=NA)
lines(ecdf(fit.zinb),verticals=T,lwd=2,col="cyan2",lty=1,pch=NA)
legend("bottomright",c("Empirical CFD","Zero-Inflated Poisson","Poisson","Quasi-Poisson","Negative Binomial","Zero-Inflated Negative Binomial")
       ,col=c("black","green","blue","gold2","red","cyan2"),lty=1,lwd=2)


################
##- Table SM1.
################
##-- Crude comparison of the mean counts and the proportion of zeros P(0),
##-- reporting 95% CI of the mean difference and the difference of the proportion of zeros.
tapply(datos$NC[datos$Study=="ET"],datos$Group[datos$Study=="ET"],mean)
t.test(datos$NC[datos$Study=="ET"]~datos$Group[datos$Study=="ET"])
x1<-prop.table(table(datos$NC[datos$Study=="ET" & datos$Group=="R"]!=0))
x2<-prop.table(table(datos$NC[datos$Study=="ET" & datos$Group=="SC"]!=0))
prop.test(rbind(t(x1),t(x2)))
tapply(datos$NC[datos$Study=="IQ"],datos$Group[datos$Study=="IQ"],mean)
t.test(datos$NC[datos$Study=="IQ" & (datos$Group=="R" | datos$Group=="SC")]~datos$Group[datos$Study=="IQ" & (datos$Group=="R" | datos$Group=="SC")])
x1<-prop.table(table(datos$NC[datos$Study=="IQ" & datos$Group=="R"]!=0))
x2<-prop.table(table(datos$NC[datos$Study=="IQ" & datos$Group=="SC"]!=0))
prop.test(rbind(t(x1),t(x2)))
datos$Group2[datos$Group=="R" | datos$Group=="S"] <- "R+S"
datos$Group2[datos$Group=="C" | datos$Group=="SC"] <- "C+SC"
datos$Group2 <- relevel(as.factor(datos$Group2),ref="R+S")
tapply(datos$NC[datos$Study=="IQ"],datos$Group2[datos$Study=="IQ"],mean)
t.test(datos$NC[datos$Study=="IQ"]~datos$Group2[datos$Study=="IQ"])
x1<-prop.table(table(datos$NC[datos$Study=="IQ" & datos$Group2=="R+S"]!=0))
x2<-prop.table(table(datos$NC[datos$Study=="IQ" & datos$Group2=="C+SC"]!=0))
prop.test(rbind(x1,x2))
datos$Group3[datos$Group=="R" | datos$Group=="C"] <- "R+C"
datos$Group3[datos$Group=="S" | datos$Group=="SC"] <- "S+SC"
datos$Group3 <- relevel(as.factor(datos$Group3),ref="R+C")
tapply(datos$NC[datos$Study=="IQ"],datos$Group3[datos$Study=="IQ"],mean)
t.test(datos$NC[datos$Study=="IQ"]~datos$Group3[datos$Study=="IQ"])
x1<-prop.table(table(datos$NC[datos$Study=="IQ" & datos$Group3=="R+C"]!=0))
x2<-prop.table(table(datos$NC[datos$Study=="IQ" & datos$Group3=="S+SC"]!=0))
prop.test(rbind(x1,x2))
datos$Group4[datos$Group=="R"] <- "R"
datos$Group4[datos$Group=="S" | datos$Group=="C" | datos$Group=="SC"] <- "C+S+SC"
datos$Group4 <- relevel(as.factor(datos$Group4),ref="R")
tapply(datos$NC[datos$Study=="IQ"],datos$Group4[datos$Study=="IQ"],mean)
t.test(datos$NC[datos$Study=="IQ"]~datos$Group4[datos$Study=="IQ"])
x1<-prop.table(table(datos$NC[datos$Study=="IQ" & datos$Group4=="R"]!=0))
x2<-prop.table(table(datos$NC[datos$Study=="IQ" & datos$Group4=="C+S+SC"]!=0))
prop.test(rbind(x1,x2))
################
##- Figure SM9.
################
##-- Mean ratio of citations (left) and odds ratio of extra-zeros (right). Empty and filled boxes represent direct and
##-- adjusted by baseline MQAI quality and length of follow up estimates. Uncertainty is estimated theoretically (Wald)
##-- and by resampling methodology (Jackknife).

#- MEAN RATIO
# ET: Adjusted ZIP (offset(FU) + Baseline Goodman scale)
S <- summary(m1 <- zeroinfl(NC ~ Goodman.Scale+offset(Follow.up)+as.factor(Group), data = datos[datos$Study=="ET",]))
estim <- c(); estim[1] <- S$coefficients$count[3,1]
for (i in 1:dim(datos[datos$Study=="ET",])[1]){d <- datos[datos$Study=="ET",][-i,]
  S <- summary(m1 <- zeroinfl(NC ~ Goodman.Scale+offset(Follow.up)+as.factor(Group), data = d))
  estim[i] <- S$coefficients$count[3,1]}
n <- dim(datos[datos$Study=="ET",])[1]; desv <-sqrt(((n-1)/n)*sum((estim-mean(estim))^2))
ET_OR1 <- exp(mean(estim)); se.Adj.ET <- desv
ET_OR1_inf <- exp(mean(estim)-qt(0.975,dim(datos[datos$Study=="ET",])[1]-1)*desv)
ET_OR1_sup <- exp(mean(estim)+qt(0.975,dim(datos[datos$Study=="ET",])[1]-1)*desv)
d1_o <- data.frame(x=c("C vs CSK"),y=round(c(ET_OR1),2),ylo=round(c(ET_OR1_inf),2),yhi=round(c(ET_OR1_sup),2))
# ET: Non-Adjusted ZIP
S <- summary(m1 <- zeroinfl(NC ~ as.factor(Group), data = datos[datos$Study=="ET",]))
estim <- c(); estim[1] <- S$coefficients$count[2,1]
for (i in 1:dim(datos[datos$Study=="ET",])[1]){d <- datos[datos$Study=="ET",][-i,]
  S <- summary(m1 <- zeroinfl(NC ~ as.factor(Group), data = d));estim[i] <- S$coefficients$count[2,1]}
n <- dim(datos[datos$Study=="ET",])[1]; desv <-sqrt(((n-1)/n)*sum((estim-mean(estim))^2))
ET_OR1 <- exp(mean(estim)); se.no.Adj.ET <- desv
ET_OR1_inf <- exp(mean(estim)-qt(0.975,dim(datos[datos$Study=="ET",])[1]-1)*desv)
ET_OR1_sup <- exp(mean(estim)+qt(0.975,dim(datos[datos$Study=="ET",])[1]-1)*desv)
d1 <- data.frame(x=c("C vs CSK"),y=round(c(ET_OR1),2),ylo=round(c(ET_OR1_inf),2),yhi=round(c(ET_OR1_sup),2))
# PLOS: Adjusted ZIP (offset(FU) + Baseline Goodman scale)
articles1 <- subset(datos,Study=="IQ" & (Group=="R" | Group=="SC"))  # Groups R vs SC
S <- summary(zeroinfl(NC ~ as.factor(Group)+offset(Follow.up)+Goodman.Scale,data=articles1))
estim <- c(); estim[1] <- S$coefficients$count[2,1]
for (i in 1:dim(articles1)[1]){d <- articles1[-i,]
  S <- summary(m1 <- zeroinfl(NC ~ as.factor(Group)+offset(Follow.up)+Goodman.Scale,data=d))
  estim[i] <- S$coefficients$count[2,1]}
n <- dim(articles1)[1]; desv <-sqrt(((n-1)/n)*sum((estim-mean(estim))^2)); PLOS_C_CSK_OR1 <- exp(mean(estim)); se.Adj.PLOS <- desv
PLOS_C_CSK_OR1_inf <- exp(mean(estim)-qt(0.975,dim(articles1)[1]-1)*desv)
PLOS_C_CSK_OR1_sup <- exp(mean(estim)+qt(0.975,dim(articles1)[1]-1)*desv)
d2_o <- data.frame(x=c("C vs CSK"),y=round(c(PLOS_C_CSK_OR1),2),ylo=round(c(PLOS_C_CSK_OR1_inf),2),yhi=round(c(PLOS_C_CSK_OR1_sup),2))
# PLOS: Non-Adjusted ZIP
S <- summary(zeroinfl(NC ~ as.factor(Group),data=articles1)); estim <- c(); estim[1] <- S$coefficients$count[2,1]
for (i in 1:dim(articles1)[1]){d <- articles1[-i,]; S <- summary(m1 <- zeroinfl(NC ~ as.factor(Group),data=d))
  estim[i] <- S$coefficients$count[2,1]}
n <- dim(articles1)[1]; desv <-sqrt(((n-1)/n)*sum((estim-mean(estim))^2)); PLOS_C_CSK_OR1 <- exp(mean(estim)); se.no.Adj.PLOS <- desv
PLOS_C_CSK_OR1_inf <- exp(mean(estim)-qt(0.975,dim(articles1)[1]-1)*desv)
PLOS_C_CSK_OR1_sup <- exp(mean(estim)+qt(0.975,dim(articles1)[1]-1)*desv)
d2 <- data.frame(x=c("C vs CSK"),y=round(c(PLOS_C_CSK_OR1),2),ylo=round(c(PLOS_C_CSK_OR1_inf),2),yhi=round(c(PLOS_C_CSK_OR1_sup),2))
# Meta-analysis
# Adjusted
ET <- rbind(d1,d1_o); PLOS_CvsCSK <- rbind(d2,d2_o)
logs <- c(log(ET$y[2]),log(PLOS_CvsCSK$y[2])); selogs <- c(se.Adj.ET,se.Adj.PLOS)
names <- c("ET study", "IQ study"); b <- data.frame(names,logs,selogs)
d <- meta.summaries(b$logs, b$selogs, names=b$names,method="fix", logscale=TRUE)
estim.Adj.meta <- round(exp(d$summary),2)
LL.estim.Adj.meta <- round(exp(d$summary-1.96*d$se.summary),2); UL.estim.Adj.meta <- round(exp(d$summary+1.96*d$se.summary),2)
# Unadjusted
logs <- c(log(ET$y[1]),log(PLOS_CvsCSK$y[1])); selogs <- c(se.no.Adj.ET,se.no.Adj.PLOS)
names <- c("ET study", "IQ study"); b <- data.frame(names,logs,selogs)
d <- meta.summaries(b$logs, b$selogs, names=b$names,method="fix", logscale=TRUE)
estim.no.Adj.meta <- round(exp(d$summary),2)
LL.estim.no.Adj.meta <- round(summary(d)$summci[1],2); UL.estim.no.Adj.meta <- round(summary(d)$summci[3],2)
# GRAPHIC - Forest plot
poin <- c(0,15); lin <- c(1,1); wid <- c(1,2); pos <- c(4:1); etiq <- c("(n=41 vs 51)","(n=27 vs 24)")
win.graph(15,8); par(oma=c(0,6,0,5),mfrow=c(1,2))
plot(NA, xlim = c(0.33,3), ylim=c(0,pos[1]),xaxt="n",yaxt="n",ylab="",xlab="",axes=F,log="x")
abline(v=1,lty=2); axis(1,c(1/3.5,1/3,0.5,1,2,3,3.5),labels=F)
mtext(c("1/3","1/2","1","2","3"),las=1,side=1,at=c(0.33,0.5,1,2,3),line=0.5)
# MA
polygon(x=c(LL.estim.Adj.meta,estim.Adj.meta,UL.estim.Adj.meta),y=c(pos[2],pos[2]+0.1,pos[2]),col="black")
polygon(x=c(LL.estim.Adj.meta,estim.Adj.meta,UL.estim.Adj.meta),y=c(pos[2],pos[2]-0.1,pos[2]),col="black")
polygon(x=c(LL.estim.no.Adj.meta,estim.no.Adj.meta,UL.estim.no.Adj.meta),y=c(pos[2]+0.5,pos[2]+0.6,pos[2]+0.5))
polygon(x=c(LL.estim.no.Adj.meta,estim.no.Adj.meta,UL.estim.no.Adj.meta),y=c(pos[2]+0.5,pos[2]+0.4,pos[2]+0.5))
# ET
segments(ET$ylo[1],pos[3],ET$yhi[1],pos[3],lwd=wid[1]); points(ET$y[1],pos[3],pch=poin[1],cex=1.5/sqrt(se.no.Adj.ET))
segments(ET$ylo[2],pos[3]-0.5,ET$yhi[2],pos[3]-0.5,lwd=wid[2]); points(ET$y[2],pos[3]-0.5,pch=poin[2],cex=1.5/sqrt(se.Adj.ET))
# IQ
segments(PLOS_CvsCSK$ylo[1],pos[4]-0.3,PLOS_CvsCSK$yhi[1],pos[4]-0.3,lwd=wid[1])
points(PLOS_CvsCSK$y[1],pos[4]-0.3,pch=poin[1],cex=1.5/sqrt(se.no.Adj.PLOS))
segments(PLOS_CvsCSK$ylo[2],pos[4]-0.7,PLOS_CvsCSK$yhi[1],pos[4]-0.7,lwd=wid[2])
points(PLOS_CvsCSK$y[2],pos[4]-0.7,pch=poin[2],cex=1.5/sqrt(se.Adj.PLOS))
# Labels
mtext("Join MA",las=2,side=2,at=pos[2]+0.5,line=4.5,font=2,cex=1.5); mtext("estimated effect",las=2,side=2,at=pos[2]+0.3,line=0,font=2,cex=1.5)
mtext("ET study",las=2,side=2,at=pos[3],line=4,font=2,cex = 1.5); mtext(etiq[1],las=2,side=2,at=pos[3]-0.2,line=2.5,cex = 1.5)
mtext("IQ study",las=2,side=2,at=pos[4]-0.3,line=4.5,font=2,cex = 1.5); mtext(etiq[2],las=2,side=2,at=pos[4]-0.5,line=2.9,cex = 1.5)
mtext("Mean Ratio (CI 95%)",las=2,side=4,at=pos[1],line=-1.1,font=2)
mtext(paste(estim.Adj.meta,"(",LL.estim.Adj.meta,";",UL.estim.Adj.meta,")"),side=4,las=2,at=pos[2],line=0.25)
mtext(paste(estim.no.Adj.meta,"(",LL.estim.no.Adj.meta,";",UL.estim.no.Adj.meta,")"),side=4,las=2,at=pos[2]+0.5,line=0.25)
mtext(paste(ET$y,"(",ET$ylo,";",ET$yhi,")"),side=4,las=2,at=c(pos[3],pos[3]-0.5),line=0.25)
mtext(paste(PLOS_CvsCSK$y,"(",PLOS_CvsCSK$ylo,";",PLOS_CvsCSK$yhi,")"),side=4,las=2,at=c(pos[4]-0.3,pos[4]-0.7),line=0.25)
mtext("Higher in intervention",las=2,side=2,at=-0.5,line=-20); mtext("Higher in control",las=2,side=2,at=-0.5,line=-4)
#- ODDS RATIO
# ET: Adjusted ZIP (offset(FU) + Baseline Goodman scale)
S <- summary(m1 <- zeroinfl(NC ~ as.factor(Group)+offset(Follow.up)+Goodman.Scale, data = datos[datos$Study=="ET",]))
estim <- c(); estim[1] <- S$coefficients$zero[2,1]
for (i in 1:dim(datos[datos$Study=="ET",])[1]){d <- datos[datos$Study=="ET",][-i,]
  S <- summary(m1 <- zeroinfl(NC ~ as.factor(Group)+offset(Follow.up)+Goodman.Scale, data = d))
  estim[i] <- S$coefficients$zero[2,1]}
n <- (dim(datos[datos$Study=="ET",])[1])-1; desv <-sqrt(((n-1)/n)*sum((estim[-81]-mean(estim[-81]))^2))
ET_OR1.0 <- exp(mean(estim)); se.Adj.ET.0 <- desv
ET_OR1_inf.0 <- exp(mean(estim)-qt(0.975,dim(datos[datos$Study=="ET",])[1]-1)*desv)
ET_OR1_sup.0 <- exp(mean(estim)+qt(0.975,dim(datos[datos$Study=="ET",])[1]-1)*desv)
d1.0_o <- data.frame(x=c("C vs CSK"),y=round(c(ET_OR1.0),2),ylo=round(c(ET_OR1_inf.0),2),yhi=round(c(ET_OR1_sup.0),2))
# ET: Non-Adjusted ZIP
S <- summary(m1 <- zeroinfl(NC ~ as.factor(Group), data = datos[datos$Study=="ET",]))
estim <- c(); estim[1] <- S$coefficients$zero[2,1]
for (i in 1:dim(datos[datos$Study=="ET",])[1]){d <- datos[datos$Study=="ET",][-i,]
  S <- summary(m1 <- zeroinfl(NC ~ as.factor(Group), data = d)); estim[i] <- S$coefficients$zero[2,1]}
n <- dim(datos[datos$Study=="ET",])[1]; desv <-sqrt(((n-1)/n)*sum((estim-mean(estim))^2))
ET_OR1.0 <- exp(mean(estim)); se.NonAdj.ET.0 <- desv
ET_OR1_inf.0 <- exp(mean(estim)-qt(0.975,dim(datos[datos$Study=="ET",])[1]-1)*desv)
ET_OR1_sup.0 <- exp(mean(estim)+qt(0.975,dim(datos[datos$Study=="ET",])[1]-1)*desv)
d1.0 <- data.frame(x=c("C vs CSK"),y=round(c(ET_OR1.0),2),ylo=round(c(ET_OR1_inf.0),2),yhi=round(c(ET_OR1_sup.0),2))
# IQ: Adjusted ZIP (offset(FU) + Baseline Goodman scale)
articles1 <- subset(datos,Study=="IQ" & (Group=="R" | Group=="SC"))  # Groups R vs SC
S <- summary(m1 <- zeroinfl(NC ~ as.factor(Group)+offset(Follow.up)+Goodman.Scale, data = articles1))
estim <- c(); estim[1] <- S$coefficients$zero[2,1]
for (i in 1:dim(articles1)[1]){d <- articles1[-i,]
  S <- summary(m1 <- zeroinfl(NC ~ as.factor(Group)+offset(Follow.up)+Goodman.Scale, data = d))
  estim[i] <- S$coefficients$zero[2,1]}
n <- dim(articles1)[1]; desv <-sqrt(((n-1)/n)*sum((estim-mean(estim))^2))
PLOS_C_CSK_OR1.0 <- exp(mean(estim)); se.Adj.PLOS.0 <- desv
PLOS_C_CSK_OR1_inf.0 <- exp(mean(estim)-qt(0.975,dim(articles1)[1]-1)*desv)
PLOS_C_CSK_OR1_sup.0 <- exp(mean(estim)+qt(0.975,dim(articles1)[1]-1)*desv)
d2.0_o <- data.frame(x=c("C vs CSK"),y=round(c(PLOS_C_CSK_OR1.0),2),ylo=round(c(PLOS_C_CSK_OR1_inf.0),2),yhi=round(c(PLOS_C_CSK_OR1_sup.0),2))
# IQ: Non-Adjusted ZIP
S <- summary(m1 <- zeroinfl(NC ~ as.factor(Group), data = articles1))
estim <- c(); estim[1] <- S$coefficients$zero[2,1]
for (i in 1:dim(articles1)[1]){d <- articles1[-i,]
  S <- summary(m1 <- zeroinfl(NC ~ as.factor(Group), data = d)); estim[i] <- S$coefficients$zero[2,1]}
n <- dim(articles1)[1]; desv <-sqrt(((n-1)/n)*sum((estim-mean(estim))^2))
PLOS_C_CSK_OR1.0 <- exp(mean(estim)); se.NonAdj.PLOS.0 <- desv
PLOS_C_CSK_OR1_inf.0 <- exp(mean(estim)-qt(0.975,dim(articles1)[1]-1)*desv)
PLOS_C_CSK_OR1_sup.0 <- exp(mean(estim)+qt(0.975,dim(articles1)[1]-1)*desv)
d2.0 <- data.frame(x=c("C vs CSK"),y=round(c(PLOS_C_CSK_OR1.0),2),ylo=round(c(PLOS_C_CSK_OR1_inf.0),2),yhi=round(c(PLOS_C_CSK_OR1_sup.0),2))
# Meta-analysis
# Adjusted
ET <- rbind(d1.0,d1.0_o); PLOS_CvsCSK <- rbind(d2.0,d2.0_o)
logs <- c(log(ET$y[2]),log(PLOS_CvsCSK$y[2])); selogs <- c(se.Adj.ET.0,se.Adj.PLOS.0)
names <- c("ET study", "IQ study"); b <- data.frame(names,logs,selogs)
d <- meta.summaries(b$logs, b$selogs, names=b$names,method="fix", logscale=TRUE)
estim.Adj.meta <- round(exp(d$summary),2)
LL.estim.Adj.meta <- round(summary(d)$summci[1],2); UL.estim.Adj.meta <- round(summary(d)$summci[3],2)
# Unadjusted
logs <- c(log(ET$y[1]),log(PLOS_CvsCSK$y[1])); selogs <- c(se.NonAdj.ET.0,se.NonAdj.PLOS.0)
names <- c("ET study", "IQ study"); b <- data.frame(names,logs,selogs)
d <- meta.summaries(b$logs, b$selogs, names=b$names,method="fix", logscale=TRUE)
estim.no.Adj.meta <- round(exp(d$summary),2)
LL.estim.no.Adj.meta <- round(summary(d)$summci[1],2); UL.estim.no.Adj.meta <- round(summary(d)$summci[3],2)
#- GRAPHIC - Forest plot
poin <- c(0,15); lin <- c(1,1); wid <- c(1,2); pos <- c(4:1); etiq <- c("(n=41 vs 51)","(n=27 vs 24)")
plot(NA, xlim = c(1/30,30), ylim=c(0,pos[1]),xaxt="n",yaxt="n",ylab="",xlab="",axes=F,log="x")
abline(v=1,lty=2); axis(1,c(1/30,1/15,1/5,1,5,15,30),labels=F)
mtext(c("1/30","1/15","1/5","1","5","15","30"),las=1,side=1,at=c(1/30,1/15,1/5,1,5,15,30),line=0.5,cex=0.8)
# MA
polygon(x=c(LL.estim.Adj.meta,estim.Adj.meta,UL.estim.Adj.meta),y=c(pos[2],pos[2]+0.1,pos[2]),col="black")
polygon(x=c(LL.estim.Adj.meta,estim.Adj.meta,UL.estim.Adj.meta),y=c(pos[2],pos[2]-0.1,pos[2]),col="black")
polygon(x=c(LL.estim.no.Adj.meta,estim.no.Adj.meta,UL.estim.no.Adj.meta),y=c(pos[2]+0.5,pos[2]+0.6,pos[2]+0.5))
polygon(x=c(LL.estim.no.Adj.meta,estim.no.Adj.meta,UL.estim.no.Adj.meta),y=c(pos[2]+0.5,pos[2]+0.4,pos[2]+0.5))
# ET
segments(ET$ylo[1],pos[3],ET$yhi[1],pos[3],lwd=wid[1]); points(ET$y[1],pos[3],pch=poin[1],cex=2.6/sqrt(se.NonAdj.ET.0))
segments(ET$ylo[2],pos[3]-0.5,ET$yhi[2],pos[3]-0.5,length=0.1); points(ET$y[2],pos[3]-0.5,pch=poin[2],cex=2.6/sqrt(se.Adj.ET.0))
# IQ
segments(PLOS_CvsCSK$ylo[1],pos[4]-0.3,PLOS_CvsCSK$yhi[1],pos[4]-0.3,lwd=wid[1])
points(PLOS_CvsCSK$y[1],pos[4]-0.3,pch=poin[1],cex=2.6/sqrt(se.NonAdj.PLOS.0))
segments(PLOS_CvsCSK$ylo[2],pos[4]-0.7,PLOS_CvsCSK$yhi[2],pos[4]-0.7,lwd=wid[2])
points(PLOS_CvsCSK$y[2],pos[4]-0.7,pch=poin[2],cex=2.6/sqrt(se.Adj.PLOS.0))
# Labels
mtext("OR (CI 95%)",las=2,side=4,at=pos[1],line=0.5,font=2)
mtext(paste(estim.Adj.meta,"(",LL.estim.Adj.meta,";",UL.estim.Adj.meta,")"),side=4,las=2,at=pos[2],line=-0.15)
mtext(paste(estim.no.Adj.meta,"(",LL.estim.no.Adj.meta,";",UL.estim.no.Adj.meta,")"),side=4,las=2,at=pos[2]+0.5,line=-0.15)
mtext(paste(ET$y,"(",ET$ylo,";",ET$yhi,")"),side=4,las=2,at=c(pos[3],pos[3]-0.5),line=-0.15)
mtext(paste(PLOS_CvsCSK$y,"(",PLOS_CvsCSK$ylo,";",PLOS_CvsCSK$yhi,")"),side=4,las=2,at=c(pos[4]-0.3,pos[4]-0.7),line=-0.15)
mtext("Higher in intervention",las=2,side=2,at=-0.5,line=-20)
mtext("Higher in control",las=2,side=2,at=-0.5,line=-5)
```
